# The HUNT lung-SNP model: genetic variants plus clinical variables improve lung cancer risk assessment over clinical models

**DOI:** 10.1007/s00432-024-05909-w

**Published:** 2024-08-12

**Authors:** Olav Toai Duc Nguyen, Ioannis Fotopoulos, Therese Haugdahl Nøst, Maria Markaki, Vincenzo Lagani, Ioannis Tsamardinos, Oluf Dimitri Røe

**Affiliations:** 1 Department of Clinical Research and Molecular Medicine, Norwegian University of Science and Technology (NTNU), Prinsesse Kristinas gate. 1, Trondheim, NO 7030 Norway; 2 Levanger Hospital, Nord-Trøndelag Hospital Trust, Cancer Clinic, Kirkegata 2, Levanger, NO 7600 Norway; 3 Department of Computer Science, University of Crete, Voutes Campus, Heraklion, GR 70013 Greece; 4 Department of Community Medicine, Faculty of Health Sciences, UiT The Arctic University of Norway, P.O. Box 6050, Langnes, Tromsø, NO-9037 Norway; 5 Department of Public Health and Nursing, Norwegian University of Science and Technology, K.G. Jebsen Center for Genetic Epidemiology, NTNU, Håkon Jarls Gate 12, Trondheim, 7030 Norway; 6 Institute of Applied and Computational Mathematics, FORTH, Heraklion, Crete, GR-700 13 Greece; 7 Biological and Environmental Sciences and Engineering Division (BESE), King Abdullah University of Science and Technology (KAUST), Thuwal, 23952 Saudi Arabia; 8 SDAIA-KAUST Center of Excellence in Data Science and Artificial Intelligence, Thuwal, 23952 Saudi Arabia; 9 Institute of Chemical Biology, Ilia State University, Tbilisi, 0162 Georgia; 10 JADBio Gnosis DA S.A, STEP-C, N. Plastira 100, Heraklion, 700-13 GR Greece; 11 Clinical Cancer Research Center, Department of Clinical Medicine, Aalborg University Hospital, Hobrovej 18-22, Aalborg, DK-9100 Denmark

**Keywords:** Lung cancer screening, HUNT Lung Cancer Model, Single nucleotide polymorphism, Lung cancer risk model, Polygenic risk score, USPSTF, NELSON, NLST

## Abstract

**Purpose:**

The HUNT Lung Cancer Model (HUNT LCM) predicts individualized 6-year lung cancer (LC) risk among individuals who ever smoked cigarettes with high precision based on eight clinical variables. Can the performance be improved by adding genetic information?

**Methods:**

A polygenic model was developed in the prospective Norwegian HUNT2 study with clinical and genotype data of individuals who ever smoked cigarettes (*n* = 30749, median follow up 15.26 years) where 160 LC were diagnosed within six years. It included the variables of the original HUNT LCM plus 22 single nucleotide polymorphisms (SNPs) highly associated with LC. External validation was performed in the prospective Norwegian Tromsø Study (*n* = 2663).

**Results:**

The novel HUNT Lung-SNP model significantly improved risk ranking of individuals over the HUNT LCM in both HUNT2 (*p* < 0.001) and Tromsø (*p* < 0.05) cohorts. Furthermore, detection rate (number of participants selected to detect one LC case) was significantly better for the HUNT Lung-SNP vs. HUNT LCM in both cohorts (42 vs. 48, *p* = 0.003 and 11 vs. 14, *p* = 0.025, respectively) as well as versus the NLST, NELSON and 2021 USPSTF criteria. The area under the receiver operating characteristic curve (AUC) was higher for the HUNT Lung-SNP in both cohorts, but significant only in HUNT2 (AUC 0.875 vs. 0.844, *p* < 0.001). However, the integrated discrimination improvement index (IDI) indicates a significant improvement of LC risk stratification by the HUNT Lung-SNP in both cohorts (IDI 0.019, *p* < 0.001 (HUNT2) and 0.013, *p* < 0.001 (Tromsø)).

**Conclusion:**

The HUNT Lung-SNP model could have a clinical impact on LC screening and has the potential to replace the HUNT LCM as well as the NLST, NELSON and 2021 USPSTF criteria in a screening setting. However, the model should be further validated in other populations and evaluated in a prospective trial setting.

**Supplementary Information:**

The online version contains supplementary material available at 10.1007/s00432-024-05909-w.

## Introduction

The NLST and NELSON studies showed that computer-tomography (CT) screening of individuals that smoke can reduce lung cancer (LC) mortality by 20–24% (Aberle et al. 2011; de Koning et al. 2020). Both studies used fixed age and smoking history criteria for screening selection. However, ¾ of people developing LC do not fulfill the NLST criteria (Pinsky and Berg 2012). To include more at-risk individuals, the US Preventive Strategy Task Force (USPSTF) introduced wider screening criteria in 2021 (Krist et al. 2021). There is no international consensus on how to best select individuals for LC screening.

Various LC clinical risk prediction models have been developed, validated, and shown performance over selection criteria used in NLST, NELSON and USPSTF (Markaki et al. 2018 Røe et al. 2019; Tammemägi et al. 2022). Several studies have tried to integrate genetic susceptibility markers to further improve their performance, but no such model has shown to be superior to clinical risk models (Chien et al. 2020; Hoggart et al. 2012; Hung et al. 2021; Li et al. 2012; Marcus et al. 2016; Qian et al. 2016; Raji et al. 2010; Spitz et al. 2013; Weissfeld et al. 2015; Young et al. 2009).

In previous work, we developed and validated the HUNT Lung Cancer Model (HUNT LCM) to predict the LC risk in individuals that ever smoked with a concordance index of 0.879 and area under the receiver operating characteristic curve (AUC) of 0.87 for a 6-year LC diagnosis (Markaki et al. 2018). It was shown to have a superior performance compared to the NLST (Markaki et al. 2018), NELSON and 2021 USPSTF criteria (Nguyen et al. 2024).

Genome-wide association studies (GWAS) have identified specific LC susceptibility regions (McKay et al. 2008, 2017). However, Single Nucleotide Polymorphisms (SNPs) alone are not predictive enough to warrant their use to identify high-risk individuals (Li et al. 2012; Qian et al. 2016). Nevertheless, SNPs carry some predictive information that could potentially increase risk prediction (Dai et al. 2019; McKay et al. 2017).

In this work, we develop and validate a new polygenic model for LC risk prediction integrating selected SNPs with the original eight clinical variables of the HUNT LCM. The performance of the new model, named HUNT Lung-SNP, is compared against the HUNT LCM, as well as the NLST, NELSON and 2021 USPSTF criteria.

## Methods

### Discovery and validation datasets

The discovery cohort was extracted from the HUNT2 study, a Norwegian prospective population study, which includes data from questionnaires, interviews, clinical measurements, and a serum biobank for all involved individuals. The HUNT2 enrolled and examined 65,240 people aged > 20 years in 1995-97 and followed up until 31.12.2011 (Krokstad et al. 2013). Genotyping information was available for 56,553 individuals, and these constitute the discovery dataset (Brumpton et al. 2022). The remaining individuals were unsuccessfully genotyped due to low blood sample quality. Missing clinical values are present in the data, with the highest percentage of missingness being in the variable “Indoor smoke exposure in hours” (17.8%, see Table 1). Missing clinical values were imputed with the median value for numerical variables or the mode for categorical variables.

The validation dataset comes from a similar population-based prospective study, the Tromsø Study (see Supplementary) (Jacobsen et al. 2012). Genotyping information was available for 6572 individuals in the Tromsø study.

### Genotyping

The DNA from the HUNT2 samples was genotyped using one of three different Illumina Human Core Exome arrays (see Supplementary). All missing values in the SNPs have been imputed. The imputation and quality control of the datasets is described in detail in the Supplementary Material. The LC associated SNPs were selected manually from the HUNT Fast-track catalogue (HUNT Fast Track GWAS catalogue) where all SNPs were associated with LC at the genome-wide significance threshold in published literature (*p* < 5 × 10^− 8^, Supplementary Table 1) by the time this study was conducted in 2018. The Tromsø cohort samples were genotyped and imputed using the same methods as described for the HUNT2 samples and the same SNPs were available in both cohorts.

### Definition of the clinical outcome

The national 11-digit personal identification number of each participant was linked to the Norwegian Cancer and Death Cause Registry. The diagnosis code of the International Classification of Diseases (ICD7) 162.1 and (ICD10) C33-34, was used to identify participants that were subsequently diagnosed with LC. Controls with a diagnosis of LC before the follow-up period were excluded. Follow up information for both the HUNT2 and Tromsø studies was obtained from the national Cancer Registry, which is updated each year. Clinical outcome was defined as “diagnosis of LC within six years” in both cohorts. Participants that develop LC within this timespan from inclusion were considered LC cases, all others were considered as controls. All cancers were clinically detected and not screen detected, and thus rarely indolent. In the survival analysis, participants that died or left the study before the six-year mark were censored. Individuals that died after LC diagnosis were considered LC associated deaths.

### Univariate analysis

The univariate association between LC and each of the original eight HUNT LCM clinical variables (sex, age, body-mass index (BMI), pack-years, number of cigarettes per day, quit time in years, hours of daily indoors smoke exposure and history of daily cough in periods through the year) was assessed through unpaired t-test (numerical variables) or chi-square test (categorical variables). The SNP genotypes were transformed into ordinal encodings as described in the literature (He et al. 2015) (see Supplementary). The association between LC and each of the 22 SNPs was evaluated through a proportional odds likelihood ratio test (Coles 2001).

### Multivariable modeling

The model for assessing LC risk was fit using the original eight HUNT LCM clinical covariates along with the 22 SNP genotype predictors. The SNP genotypes were transformed into ordinal encodings as described in the Supplementary. The outcome was defined as mentioned above, “diagnosis of LC within six years.” To establish the final model, we use a shrinkage methodology (Steyerberg et al. 2001), which relies on refitting the logistic regression model 100 times, each time over resampled data. Through this bootstrapping process we estimate to what extent the coefficients of the original logistic models should be shrunk. This methodology has shown to decrease the probability of overfitting (Steyerberg et al. 2001), and is described in more detail in the Supplementary Material.

### Model validation

**Fig. 1:**
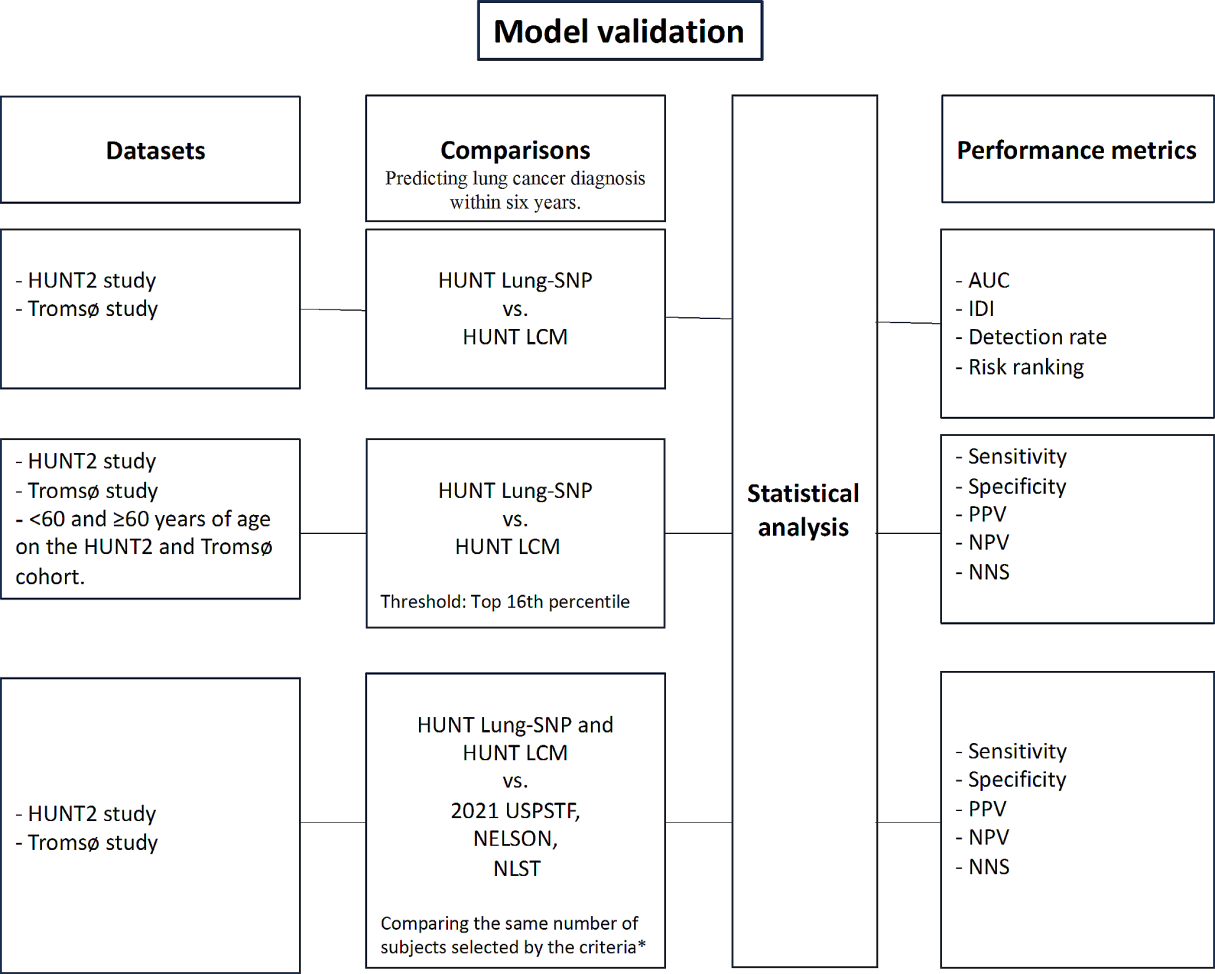
Model validation. Model validation of the HUNT Lung-SNP model against the HUNT Lancer Model (HUNT LCM) and the criteria 2021 USPSTF, NELSON and NLST on the datasets of HUNT2 and Tromsø study. *For a fair comparison, a risk threshold selecting the same number to screen as the USPSTF 2021, NELSON and NLST criteria as a benchmark was used. AUC, area under the receiver operating characteristic curve; IDI, integrated discrimination improvement index; NPV, negative predictive value; PPV, positive predictive value; NNS, number needed to screen to identify one case of lung cancer

The validation of the HUNT Lung-SNP model was performed as shown in Fig. 1.

Sample-level risk scores provided by the HUNT Lung-SNP were contrasted against the predictions provided by the original HUNT LCM (algorithm in Supplementary Appendix page 5 in Markaki et al. (2018)), both on the HUNT2 and Tromsø cohort. The AUC, integrated discrimination improvement index (IDI), detection rate (number of individuals needed to screen, NNS, to detect/predict one LC case) and ranking of risk were used as performance metrics. Statistical significance of the differences was assessed through non-parametric statistical tests (see Supplementary) (DeLong et al. 1988; Kang et al. 2015). Calibration, agreement between predicted and observed LC cases in the cohorts, was evaluated by predictiveness curve (Markaki et al. 2018).

To stratify individuals in high- and low-risk categories according to the HUNT Lung-SNP and HUNT LCM risk scores, a cut-off for each model was derived corresponding to the top 16th percentile of their respective in-sample predictions. The cutpoint of top 16th percentile was chosen according to recommendations from Royston et al. (Markaki et al. 2018; Royston and Altman 2013). The two models were then compared both on the HUNT2 and Tromsø cohort according to sensitivity, specificity, positive predictive value (PPV) and negative predictive value (NPV). The statistical significance for differences in these metrics was assessed through a permutation-based test (see Supplementary). Furthermore, to analyze whether the two models perform differently depending on the age of the population, a comparison was performed on the subpopulations < 60 and ≥ 60 years of age on the HUNT2 and Tromsø cohort.

For both models we used the Kolmogorov-Smirnov test to contrast participants’ risk ranking versus the cumulative number of LC diagnosed.

We further contrasted both the HUNT LCM and HUNT Lung-SNP against the NLST, NELSON and 2021 USPSTF criteria sets for LC diagnosis within six years. To be able to compare risk assessment models, a risk threshold resulting in the same number of participants to screen was set. Given this threshold, we present several metrics of predictive performance. The statistical significance of differences is assessed using a permutation-based, non-parametric approach (see Supplementary).

Finally, the overall survival between the sub-cohorts selected by the HUNT Lung-SNP, NELSON and 2021 USPSTF criteria was investigated. The survival was calculated as survival from LC diagnosis in the cases, and from the time of inclusion to time of death of the LC high-risk versus low-risk individuals. Kaplan-Meier curves were used to visualize survival functions, while the log-rank test was used to evaluate statistical significance in their differences. For all analyses *p* < 0.05 was used as the statistical significance level. The R Statistical Software version 4.2.1 (2022-06-23) was used to perform the analyses.

### Cost of implementing a SNP analysis

The scenario used in calculating additional cost was to apply the HUNT Lung-SNP model on all participants who ever smoked in the HUNT cohort. We calculated the cost of the SNP-analysis in terms of cost per quality-adjusted life year (QALY), to assess whether incorporating genetic variants in a LC prediction model is cost-effective. This analysis was performed using estimates of (a) the current administrative costs related to blood drawing, (b) the cost of the genetic analysis of the 22 SNPs, (c) the average years life lost (YLL) per LC case, and (d) the health-related quality of life (HRQL) score for LC.

### Role of the funding sources

The funding sources had no role in study conception, design, data interpretation, writing of the report, or decision to submit the paper for publication.

## Results

### SNPs characteristics

Among the 22 SNPs selected, one SNP was associated with small-cell LC, four with squamous cell carcinoma, six with lung adenocarcinoma and 13 with lung carcinoma (Supplementary Table 1). Furthermore, all 22 SNPs have been found significant in one or more major ethnic groups, including Latin American, African American, Caucasian, and Asian (one, three, 12 and 16 SNPs, respectively) (Supplementary Table 1, Supplementary Fig. 1).

### Discovery (HUNT2) and validation (Tromsø) cohorts

The discovery cohort comprised 30,749 genotyped individuals that ever smoked with near complete data on the HUNT LCM clinical variables (Table 1). Among these a total of 2366 was censored. After six years of follow-up, 160 had been diagnosed with LC. In univariate analysis, all of the eight clinical variables and six of the 22 SNPs were significantly associated with LC occurrence within six years (Table 1, Supplementary Table 1). Most of the included participants had all HUNT LCM clinical variables measured at enrollment: sex, age, BMI, pack-years, number of cigarettes per day, quit time, hours of daily indoors smoke exposure and cough in periods through the year (Table 1).

**Table 1:** Descriptive statistics for the discovery (HUNT2) and validation cohort (Tromsø)

	Discovery cohort (HUNT2)				Validation cohort (Tromsø)			
Clinical variables	*N*	No lung cancer *N* = 30,589	Lung cancer *N* = 160	*P*-value	*N*	No lung cancer *N* = 2624	Lung cancer *N* = 39	*P*-value
**Sex** - Female - Male	30,749 (100%)	14,688 (52.0%) 15,901 (48.0%)	55 (34.4%) 105 (65.6%)	< 0.001	2663	1338 (51.0%) 1286 (49.0%)	17 (43.6%) 22 (56.4%)	0.359
**Age** - Mean (SD) - Range	30,749 (100%)	51.218 (15.147) 20.2-100.3	66.589 (9.665) 40.5–89.9	< 0.001	2663	49.862 (12.268) 25.0–81.0	71.128 (5.569) 59.0–82.0	< 0.001
**Pack-years** - Mean (SD) - Range	27,724 (90.2%)	13.431 (11.378) 0.0-165.0	27.069 (13.898) 2.4–106.0	< 0.001	2663	13.161 (12.225) 0.0-120.0	24.242 (19.390) 3.0–90.0	< 0.001
**Daily cough parts of the year** - No - Yes	30,713 (99.9%)	24,812 (81.1%) 5741 (18.8%)	97 (60.6%) 63 (39.4%)	< 0.001	2663	2136 (81.4%) 488 (18.6%)	28 (71.8%) 11 (28.2%)	0.127
**Indoor smoke exposure in hours** - Mean (SD) - Range	25,272 (82.2%)	2.513 (4.185) 0.0–24.0	3.766 (5.018) 0.0–18.0	0.002	2663	2.744 (4.000) 0.0–24.0	4.256 (5.369) 0.0–24.0	0.020
**Quit time in years** - Mean (SD) - Range	29,754 (96.8%)	6.957 (10.541) 0.0–75.0	3.731 (8.378) 0.0–40.0	< 0.001	2663	6.132 (9.927) 0.0–76.0	4.846 (7.799) 0.0–30.0	0.421
**Cigarettes daily** - Mean (SD) - Range	27,976 (91%)	11.709 (6.743) 1.0–70.0	12.971 (7.563) 1.0–60.0	0.028	2663	11.653 (7.189) 1.0–70.0	12.654 (7.054) 3.0–40.0	0.388
**Body Mass Index (BMI)** - Mean (SD) - Range	30,589 (99.5%)	26.257 (4.029) 14.9–52.8	25.251 (3.883) 17.1–36.7	0.002	2663	25.668 (3.747) 15.8–44.4	25.908 (4.298) 17.6–38.3	0.692

Descriptive statistics for the discovery (HUNT2) and validation cohort (Tromsø). All participants were individuals that ever smoked cigarettes. The statistical association of each variable with lung cancer diagnosis within six years (*p* < 0.05). In the HUNT2 cohort the missing values were imputed. In the Tromsø cohort the participants with all variables intact were selected

Among the 6572 individuals genotyped in the Tromsø study, five never smoked, 1197 lacked smoking information, while 2707 lacked one or more of the HUNT LCM variables, leading to the inclusion of 2663 individuals who ever smoked with complete data. Among these, two were censored and 39 were diagnosed with LC within the six-year follow-up. Three of the eight clinical variables and three SNPs were significantly associated with LC in univariate analysis (Table 1, Supplementary Table 2).

### 22 SNPs model

A logistic regression model based on the 22 SNPs alone showed a predictive power with an AUC of 0.625 (95% CI 0.583–0.666) in the HUNT2 population, discovery dataset (Supplementary Table 3).

### Contrasting HUNT Lung-SNP and HUNT LCM

In the HUNT2 cohort, the HUNT Lung-SNP outperformed the HUNT LCM in terms of ranking HUNT2 participants with respect to their risk of developing LC within six-years: AUC 0.875 (95% confidence interval (CI) 0.854–0.896) vs. 0.844 (95% CI 0.820–0.869), *p* < 0.001. In the validation cohort, the HUNT Lung-SNP also performed better than the HUNT LCM, albeit not statistically significant, AUC 0.916 (95% CI 0.880–0.948) vs. 0.876 (95% CI 0.823–0.921), *p* = 0.086 (Table 2). Furthermore, the IDI between the two models indicates that the HUNT Lung-SNP significantly improve the LC risk stratification compared to the original HUNT LCM with an IDI of 0.019 (95% CI 0.015–0.025), *p* < 0.001 and of 0.013 (95% CI 0.008–0.018), *p* < 0.001 in the HUNT2 and Tromsø cohorts, respectively. Calibration was adequate for both models with predicted risk close to observed risk in both cohorts (Supplementary Fig. 2).

**Table 2:** HUNT Lung-SNP and HUNT LCM performances in predicting lung cancer within six years

	HUNT LCM	HUNT Lung-SNP	*P*-value
	AUC (95% CI)	AUC (95% CI)	
**Discovery (HUNT2)**, *** N* = 30,749**	0.844 (0.820–0.869)	0.875 (0.854–0.896)	< 0.001
**Validation (Tromsø)**, * N*** = 2663**	0.876 (0.823–0.921)	0.916 (0.880–0.948)	0.086

HUNT Lung-SNP and HUNT LCM performances in predicting lung cancer within six years

Evaluations performed on the discovery (HUNT2) and validation (Tromsø) cohort, for each evaluation the Receiver Operating Characteristics (ROC) Area Under the Curve (AUC) are reported. CI, confidence interval

**Fig. 2:**
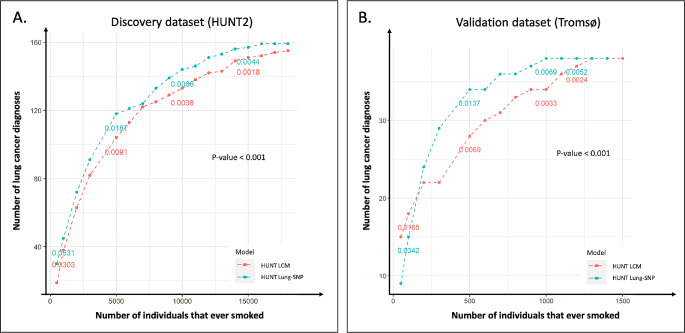
Comparison of risk ranking between the HUNT LCM and HUNT Lung-SNP model. Comparison of ranking of individuals that ever smoked by risk score in the prospective population-based HUNT2 and Tromsø studies applying the HUNT LCM and HUNT Lung-SNP model and their capacity to identify individuals that will develop lung cancer within six years. Individuals are ranked from highest to lowest risk according to the respective model from left to right (x-axis). The cumulative number of diagnosed lung cancer is reported on the y-axis. **(A)** In the HUNT2 population there are *n* = 30,749 individuals that ever smoked and *n* = 160 lung cancers diagnosed in six years. **(B)** In the Tromsø population there are *n* = 2663 individuals that ever smoked and *n* = 39 lung cancers diagnosed in six years. Comparison of distributions by the Kolmogorov-Smirnov test, *p* < 0.05 for both cohorts

Ranking of individuals according to risk score (potential screenees) versus the cumulative number of LC diagnosed for the two models, showed that the HUNT Lung-SNP’s performance improved significantly compared to the HUNT LCM in both the HUNT2 (*p* < 0.001) and Tromsø cohort (*p* < 0.05) (Fig. 2).

**Fig. 3:**
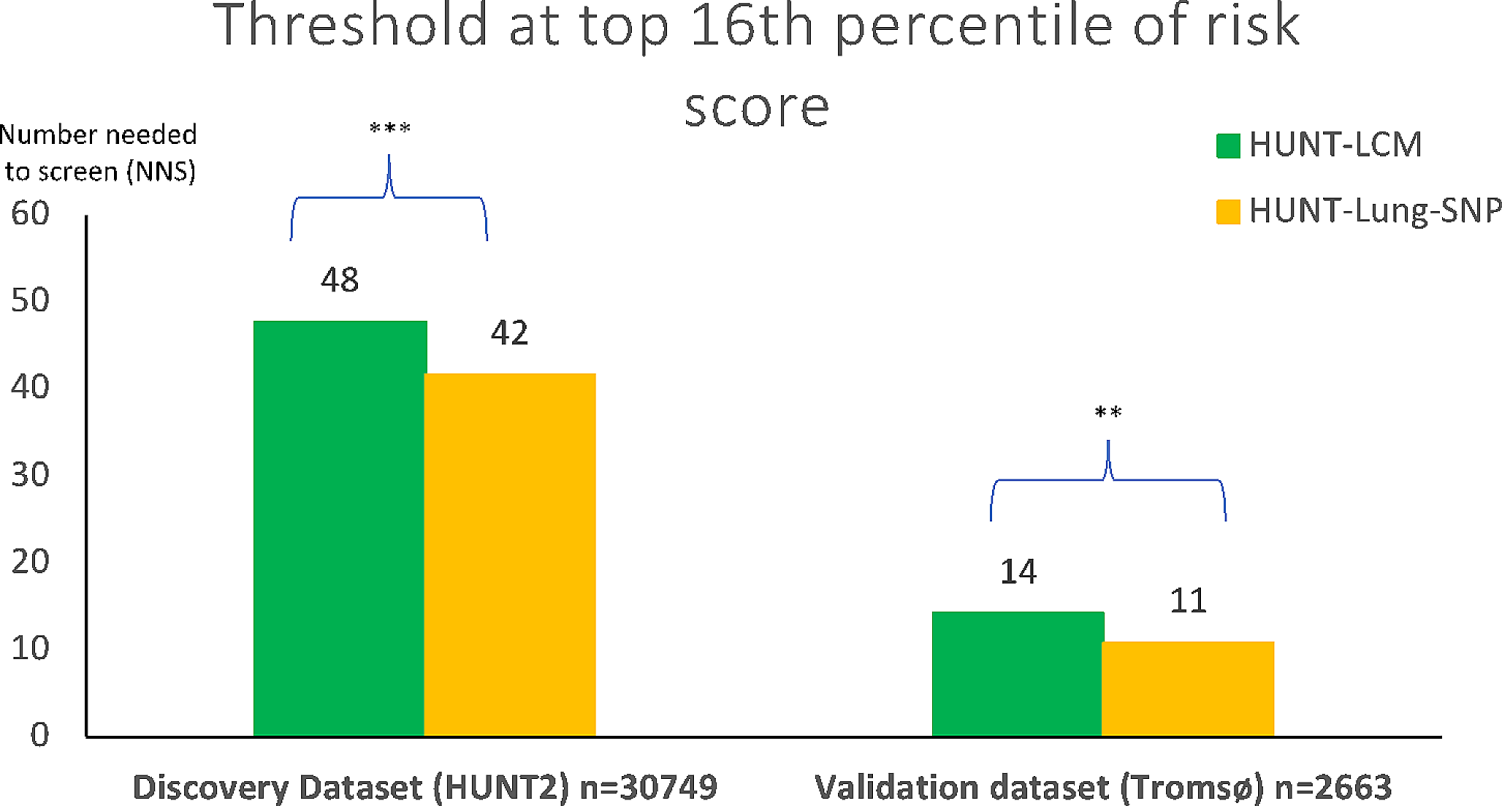
Number needed to screen (NNS). NNS to identify one case of lung cancer in the HUNT2 and Tromsø population. NNS computed when the threshold is set at the top 16th percentile of risk score. *p < 0.05. **p < 0.01

When individuals were stratified as having high or low risk according to the top 16th or bottom 84th percentile risk score, respectively, the HUNT Lung-SNP model showed increased performance across all metrics on both cohorts (Supplementary Table 4). In the HUNT2 cohort there was a significant gain in sensitivity (73.75% vs. 64.38%, *p* = 0.001), PPV (2.40% vs. 2.09%, *p* = 0.004) and NPV (99.84% vs. 99.78%, *p* < 0.001) while specificity was higher, albeit not significant. In the Tromsø cohort, sensitivity (76.92% vs. 61.54%, *p* = 0.15) and specificity (88.76% vs. 87.92%) differences were numerically even larger, albeit not statistically significant. The PPV (6.91% vs. 5.69%, *p* = 0.026) and NPV (99.62% vs. 99.35%, *p* = 0.041) were significantly different in the Tromsø cohort. Furthermore, the detection or prediction rate, defined as the number of individuals needed to screen (NNS) to detect one LC case on average, was significantly lower for the HUNT Lung-SNP compared to HUNT LCM (Fig. 3), both in the HUNT2 (NNS of 42 vs. 48, *p* = 0.003) and Tromsø cohort (NNS of 11 vs. 14, *p* = 0.025).

When the HUNT2 cohort was split by age into subpopulations of < 60 (*n* = 21,762) and ≥ 60 (*n* = 8987) years of age, the HUNT Lung-SNP achieved the same sensitivity (45.95% vs. 45.95%, *p* = 0.625) but a higher specificity (95.66% vs. 93.84%, *p* < 0.001) in the younger population (< 60 years) compared to the HUNT LCM, and better detection rate (NNS of 56 vs. 80, *p* < 0.001). For the older participants (≥ 60 years), the HUNT Lung-SNP achieved higher sensitivity (82.11% vs. 69.92%, *p* < 0.001) but with a lower specificity (56.45% vs. 60.75%, *p* < 0.001) than the HUNT LCM, and similar detection rate (NNS of 39 vs. 41, *p* = 0.273) (Supplementary Table 5).

### Contrasting the HUNT Lung-SNP and HUNT LCM against the NLST, NELSON and USPSTF criteria

In the HUNT2 cohort, when selecting the same number of high-risk individuals as the NLST, NELSON and 2021 USPSTF criteria, the HUNT Lung-SNP outperformed all the three criteria in terms of number of detected LC and corresponding sensitivities (*p* < 0.01). Similar results were found with the HUNT LCM (Supplementary results, Supplementary Tables 6, 7, and 8).

In terms of NNS to identify one LC case, the HUNT Lung-SNP was the most well-performing model in the HUNT2 cohort, with NNS of 24 vs. 40 (NLST), 31 vs. 53 (NELSON) and 39 vs. 51 (USPSTF), *p* < 0.01 for all comparisons. Similar findings were found with HUNT LCM (Supplementary Fig. 3A-C).

By applying the top 16th percentile as a cutoff for risk stratification the HUNT Lung-SNP identified **≈** 280%, 168% and 50% more cases in the HUNT2 individuals that ever smoked in six years compared to the NLST, NELSON and USPSTF criteria, respectively (Supplementary Fig. 4).

Similar significant results were found in the Tromsø cohort for both the HUNT Lung-SNP and HUNT LCM, except for the HUNT Lung-SNP under the application of NLST criteria, where the increased number of detected LC and the corresponding sensitivity, as well as the lower NNS did not reach statistical significance compared to the NLST criteria (Supplementary results).

### Survival analysis

The survival analysis showed non-significant differences in median survival from diagnosis of participants that developed LC within six years predicted by the HUNT Lung-SNP compared to the NLST, NELSON and USPSTF criteria (Supplementary Fig. 5).

### Cost of SNP analysis

The administrative costs related to blood drawing in our public hospital is estimated to $14 USD per blood test, while the cost of the genetic analysis of the SNP-panel including all the 22 SNPs can be estimated to $23 USD per analysis (Illumina 2023). Using the top 16th percentile, the HUNT Lung-SNP predicted 21 unique cases in six years (Supplementary Tables 9 and Supplementary Fig. 6) and the HUNT LCM predicted 6 unique cases, thus 15 surplus LC cases were predicted by the SNP model. Based on previous publications (Brustugun et al. 2014; Burnet et al. 2005), an average of YLL per LC case is estimated to be 15 years, given survival from LC. However, it is unlikely that all LC cases will survive from LC. Based on a 5-year relative survival rate of 68% on LC stage I (Norway 2023), an average YLL per LC case of 10 years was applied. A health-related quality of life (HRQL) score of 0.75 (Behar Harpaz et al. 2023) (meaning that 3/4 of the time saved represents life in full health) was applied.

Based on the price estimates, the YLL and the HRQL score, the additional cost will be $1,137,713 USD if the SNP-analysis is performed on all individuals that ever smoked in the HUNT cohort (*n* = 30,749). By factoring in all data, the cost of the SNP-analysis will be $10,113 USD/QALY.

## Discussion

This study shows that the HUNT Lung-SNP performs significantly better in ranking individuals by risk and decreases the number needed to screen compared to the HUNT LCM, NLST, NELSON and USPSTF criteria. To our knowledge, the HUNT Lung-SNP is the first risk model where adding genetic information improves LC risk assessment for high-risk individuals over a validated risk model and over several clinical criteria.

### HUNT Lung-SNP against HUNT LCM

Risk ranking is essential for defining the performance of a risk model. Here we found a significant improvement of risk ranking in the polygenic risk model over the clinical model, both in the discovery and validation cohort (Fig. 2). This translated into a lower NNS, an important metric for evaluating the effectiveness of potential screening. Specifically, we computed the average number of screenings to detect one LC (NNS) in the ranked list of risk according to each model. The NNS is significantly better in the HUNT Lung-SNP versus HUNT LCM and all the clinical screening criteria tested. This result indicates that the HUNT Lung-SNP could have a clinical impact in LC screening and replace the HUNT LCM and be an alternative to the clinical criteria in screening settings.

Polygenic risk score based on LC associated SNPs seems to have an independent risk stratification beyond age and smoking history (Dai et al. 2019). However, no LC risk model, based only on genetic information, has shown sufficient performance for clinical use, let alone for screening purposes (Li et al. 2012; Young et al. 2009). This is consistent with our findings where the 22 SNPs model alone had a modest AUC of 0.625 (95% CI 0.583–0.666). Several groups have added LC-associated genetic variants identified in GWAS to LC risk assessment models attempting to improve the models, but so far with disappointing results (Hung et al. 2021; Li et al. 2012; Qian et al. 2016; Young et al. 2009). Most of these studies were without external validation, all were case-control studies except for one prospective-based study (Supplementary Table 10). Although adding genetic information to risk models has shown limited impact on a risk model’s risk assessment so far, Hung et al. did observed that genetic information could be informative regarding assessing the individual’s age for reaching the low dose CT screening-eligible threshold (Hung et al. 2021). However, the study from Hung et al. lacked external validation, only performed a split-sample validation and validation in an external cohort without genetic information on each subject (Hung et al. 2021). To our knowledge, the present study is the first externally validated, prospective cohort study showing that genetic information can significantly improve LC risk assessment compared to a validated risk model in terms of risk ranking and detection rate.

### HUNT Lung-SNP and HUNT LCM against the NLST, NELSON and USPSTF criteria

The HUNT Lung-SNP outperformed the 2021 USPSTF and NELSON criteria in both HUNT2 and Tromsø cohort when selecting the same number as the two criteria, respectively. When selecting the same number as the NLST criteria the HUNT Lung-SNP performs significantly better in the HUNT2 cohort, but not in the validation cohort (see Supplementary). This could be due to the low number of participants in the Tromsø cohort combined with the strict criteria of the NLST compared to the USPSTF and NELSON, resulting in a lower number of individuals selected by the NLST (*n* = 101) in the validation cohort (see Supplementary). The HUNT LCM showed similar results in both cohorts, but with less numerically detected LC and higher NNS compared to HUNT Lung-SNPs, except when selecting the same number as the NLST criteria (see Supplementary).

### HUNT Lung-SNP model in subgroups

Younger individuals that smoke and individuals with low number of pack-years are not eligible for LC screening according to current guidelines. It is known that certain genetic predispositions have been associated with increased risk of early onset (< 51 years) LC independently of heavy smoking (Timofeeva et al. 2010). In line with this, Hung et al. reported that genetic information contributed to their risk model in those with younger age onset (< 51 years), albeit the AUC increased only moderately in their study with genetic information compared without (Hung et al. 2021). Our analysis of the HUNT2 cohort supports this, showing a significantly lower number of screenings needed per cancer detected (NNS of 56 vs. 80) for the HUNT Lung-SNP versus HUNT LCM in the younger participants (< 60 years) (Supplementary Table 5). This needs further validation since only two cases were below 60 years of age when included in the Tromsø cohort. Moreover, most of the patients predicted by the HUNT Lung-SNP but missed by the HUNT LCM, had very low number of smoking pack-years, as low as two pack-years but still reached a high risk score (Supplementary Table 9). This indicates an important role of incorporating SNPs for prediction in groups where the clinical risk model is not effective.

### Discrimination power between the HUNT Lung-SNP and HUNT LCM

The numerical AUC differences between the HUNT Lung-SNP and HUNT LCM are arguably small, and in the validation cohort they do not reach statistical significance. However, this is probably because the AUC is computed as averages over all individuals, including a large portion of the population with very low LC risk. Furthermore, concerns have been raised on AUC ability to capture the incremental value of new markers in risk prediction in a clinical meaningful way (Kerr et al. 2011). The IDI has been proposed as a complementary to AUC in measuring the discrimination improvement (Kerr et al. 2011). The IDI between the HUNT Lung-SNP and HUNT LCM indicates a significant improvement of LC risk stratification by the HUNT Lung-SNP. Furthermore, when we examined the behavior of the models in the high-risk populations (e.g. top 16th percentile risk score), the differences of the models were more apparent.

### Cost and feasibility

The approximate analysis of cost of SNP testing and cost-effectiveness was performed and showed that adding genetic test in a LC model requires some more resources than the clinical model, but still within what is both feasible and cost-effective. We found that the cost of the SNP-analysis per QALY could be $10,113 USD/QALY, which is far below the cost per QALY threshold set by many high income countries, e.g. NICE for England and Wales has set the cost per QALY threshold between £20,000 and £30,000 (=$25,000–38,000 USD) (Office for Health Improvement and Disparities 2020), the United States has set it at $50,000-100,000 USD (Ubel et al. 2003), and Norway 275,000-825,000 NOK (=$25,000–77,000 USD) (Magnussen 2015; Norheim et al. 2014; Ottersen et al. 2016). It should be noted that we expect that the cost of genetic tests to drop in the future (Wetterstrand 2021), becoming even more accessible. We emphasize that this is a simple cost-effectiveness calculation and that a comprehensive analysis using more detailed assumptions will be the focus of future research.

Finally, one can envision methods for optimizing the selection of patients for SNP analysis, e.g., using the clinical and SNP model successively. We plan to explore these approaches in future studies.

### Strengths and limitations

There are several strengths to this study: (1) The prospective study design of both cohorts. (2) The sample size of the HUNT2 cohort, the long follow-up time, and high-quality clinical data of apparently healthy individuals in a population. The validation cohort was smaller, but compared to previous reported studies the variables and SNPs matched the qualities of the HUNT cohort closely. (3) The SNPs were analyzed in high-quality high-throughput platforms at centralized University facilities. (4) All cancers were clinically detected, and thus rarely indolent, in contrast to many screen-detected cancers (Esserman et al. 2014), where about 9% of screen-detected LC have been estimated to be indolent (de Koning et al. 2020). Results from our survival analysis supports that the HUNT Lung-SNP do identify individuals with high risk of non-indolent LC (Supplementary Fig. 5). (5) As far as we know, this is the first study where the SNPs in a risk model are associated with all the three main histological subgroups of LC: adenocarcinoma, squamous cell carcinoma and small-cell LC (Supplementary Table 1).

The HUNT Lung-SNP model, besides its predictive power, has also some apparent strengths over other models. (1) All the clinical variables in the HUNT LCM and SNP model are easily retrieved from the individuals’ memory and are not dependent on culture-specific or diagnosis-based factors as e.g. in PLCO_m2012_ (education, ethnicity, history of COPD or family history of LC) (Røe et al. 2019). However, we acknowledge that the two variables “symptoms of daily cough in periods of the year” and “hours of indoor smoke” are not as easily to answer accurately as the rest of the clinical variables in the model, and these two are often unavailable in databases from other countries. If neither of these variables are available, one may use the HUNT LCM omitting these two, or our previously published model, the “Reduced” HUNT model(Røe et al. 2019). (2) The relatively easy assessment of genetic information with three possible genotype combinations (homozygous for the reference, heterozygous or homozygous for the alternative allele) compared to other molecular components such as proteins or microRNAs. (3) Only one blood test is needed as SNPs do not change throughout life. (4) The SNPs included have been found significant in one or more major ethnic groups (Supplementary Table 1, Supplementary Fig. 1), which can indicate validity in global populations, but could need recalibration for certain populations.

There are some limitations to be aware of: (1) Susceptibility polymorphisms identified in GWASs can vary in different ethnic populations. The HUNT Lung-SNP has only been externally validated in Scandinavian populations. (2) By the time this study was conducted in 2018, only 22 LC associated SNPs (*p* < 5 × 10^− 8^) were available in the HUNT Fast-track catalogue (HUNT Fast Track GWAS catalogue), knowledge has evolved, and far more genome-wide significant (*p* < 5 × 10^− 8^) LC associated SNPs have been identified since then (Long et al. 2022). (3) Our dataset is affected by class imbalance, with a proportion between the number of events and the number of variables (events per variable proportion, EPV) of three, quite below the recommended value EPV ≥ 10 (Steyerberg and Vergouwe 2014). The strategy of shrinking coefficients through bootstrapping was adopted during the training of the HUNT Lung-SNP to mitigate the issue of class imbalance while regulating the overestimation on the predictions (see Methods). (4) We recognize a potential bias issue in the validation cohort due to filtering out incomplete data, resulting in only 2663 out of 6572 individuals being included in the analysis of the Tromsø data.

## Conclusions

In conclusion, our research demonstrates for the first time that a polygenic risk prediction model for LC combining clinical variables with SNP can significantly improve the performance of LC risk ranking and NNS over a validated clinical model, HUNT LCM, and over current clinical criteria. Thus, we believe that risk stratification using the HUNT Lung-SNP model followed by annual CT lung screening is feasible and would substantially reduce the over- and underdetection rate compared with the CT LC screening model based on the NLST, NELSON or 2021 USPSTF criteria. Our results support that the HUNT Lung-SNP model should be validated in populations of various ethnicities and subgroups (younger individuals that smoke and individuals with few pack-years), and tested prospectively in screening studies or programs.

## Electronic supplementary material

Below is the link to the electronic supplementary material.

Supplementary Material 1

## Data Availability

In agreement with the license agreements applicable to this study, only the named authors were given full access to the data during the study. This is to ensure that all personal and health information of the participants in the HUNT and Tromsø studies is kept confidential. Detailed information about accessing the HUNT and Tromsø studies are available on the website of the HUNT study (https://www.ntnu.edu/hunt) and Tromsø study (https://uit.no/research/tromsostudy).

## References

[CR1] Aberle DR, Adams AM, Berg CD, Black WC, Clapp JD, Fagerstrom RM et al (2011) Reduced lung-cancer mortality with low-dose computed tomographic screening. N Engl J Med 365:395–409. 10.1056/NEJMoa1102873 21714641 10.1056/NEJMoa1102873 PMC4356534

[CR2] Behar Harpaz S, Weber MF, Wade S, Ngo PJ, Vaneckova P, Sarich PEA et al (2023) Updated cost-effectiveness analysis of lung cancer screening for Australia, capturing differences in the health economic impact of NELSON and NLST outcomes. Br J Cancer 128:91–101. 10.1038/s41416-022-02026-8 36323879 10.1038/s41416-022-02026-8 PMC9814515

[CR3] Brumpton BM, Graham S, Surakka I, Skogholt AH, Løset M, Fritsche LG et al (2022) The HUNT study: a population-based cohort for genetic research. Cell Genom 2:100193. 10.1016/j.xgen.2022.100193 36777998 10.1016/j.xgen.2022.100193 PMC9903730

[CR4] Brustugun OT, Møller B, Helland A (2014) Years of life lost as a measure of cancer burden on a national level. Br J Cancer 111:1014–1020. 10.1038/bjc.2014.364 24983370 10.1038/bjc.2014.364 PMC4150272

[CR5] Burnet NG, Jefferies SJ, Benson RJ, Hunt DP, Treasure FP (2005) Years of life lost (YLL) from cancer is an important measure of population burden–and should be considered when allocating research funds. Br J Cancer 92:241–245. 10.1038/sj.bjc.6602321 15655548 10.1038/sj.bjc.6602321 PMC2361853

[CR6] Chien LH, Chen CH, Chen TY, Chang GC, Tsai YH, Hsiao CF et al (2020) Predicting Lung Cancer Occurrence in Never-Smoking Females in Asia: TNSF-SQ, a Prediction Model. Cancer epidemiology, biomarkers & prevention: a publication of the American Association for Cancer Research, cosponsored by the American Society of Preventive Oncology. ;29:452-9. 10.1158/1055-9965.Epi-19-1221 10.1158/1055-9965.EPI-19-1221 31848206

[CR7] Coles S (2001) An introduction to statistical modeling of extreme values. Springer-, London, U.K.

[CR8] Dai J, Lv J, Zhu M, Wang Y, Qin N, Ma H et al (2019) Identification of risk loci and a polygenic risk score for lung cancer: a large-scale prospective cohort study in Chinese populations. Lancet Respir Med 7:881–891. 10.1016/s2213-2600(19)30144-4 31326317 10.1016/S2213-2600(19)30144-4 PMC7015703

[CR9] de Koning HJ, van der Aalst CM, de Jong PA, Scholten ET, Nackaerts K, Heuvelmans MA et al (2020) Reduced lung-Cancer mortality with volume CT screening in a Randomized Trial. N Engl J Med 382:503–513. 10.1056/NEJMoa1911793 31995683 10.1056/NEJMoa1911793

[CR10] DeLong ER, DeLong DM, Clarke-Pearson DL (1988) Comparing the areas under two or more correlated receiver operating characteristic curves: a nonparametric approach. Biometrics 44:837–845 3203132

[CR12] Esserman LJ, Thompson IM, Reid B, Nelson P, Ransohoff DF, Welch HG et al (2014) Addressing overdiagnosis and overtreatment in cancer: a prescription for change. Lancet Oncol 15:e234–e242. 10.1016/s1470-2045(13)70598-9 24807866 10.1016/S1470-2045(13)70598-9 PMC4322920

[CR13] He D, Wang Z, Parida L (2015) Data-driven encoding for quantitative genetic trait prediction. BMC Bioinformatics 16:S10. 10.1186/1471-2105-16-S1-S10 25707435 10.1186/1471-2105-16-S1-S10 PMC4571493

[CR14] Hoggart C, Brennan P, Tjonneland A, Vogel U, Overvad K, Østergaard JN et al (2012) A risk model for lung cancer incidence. Cancer Prev Res (Philadelphia Pa) 5:834–846. 10.1158/1940-6207.Capr-11-0237 10.1158/1940-6207.CAPR-11-0237 PMC4295118 22496387

[CR15] Hung RJ, Warkentin MT, Brhane Y, Chatterjee N, Christiani DC, Landi MT et al (2021) Assessing Lung Cancer Absolute Risk Trajectory based on a polygenic risk model. Cancer Res 81:1607–1615. 10.1158/0008-5472.Can-20-1237 33472890 10.1158/0008-5472.CAN-20-1237 PMC7969419

[CR16] Illumina, Cost of Next-Generation Sequencing (2023). Illumina. https://emea.illumina.com/science/technology/next-generation-sequencing/beginners/ngs-cost.html. Accessed 15 Sept 2023

[CR17] Jacobsen BK, Eggen AE, Mathiesen EB, Wilsgaard T, Njølstad I (2012) Cohort profile: the Tromso Study. Int J Epidemiol 41:961–967. 10.1093/ije/dyr049 21422063 10.1093/ije/dyr049 PMC3429870

[CR18] Kang L, Chen W, Petrick NA, Gallas BD (2015) Comparing two correlated C indices with right-censored survival outcome: a one-shot nonparametric approach. Stat Med 34:685–703. 10.1002/sim.6370 25399736 10.1002/sim.6370 PMC4314453

[CR19] Kerr KF, McClelland RL, Brown ER, Lumley T (2011) Evaluating the incremental value of new biomarkers with Integrated discrimination improvement. Am J Epidemiol 174:364–374. 10.1093/aje/kwr086 21673124 10.1093/aje/kwr086 PMC3202159

[CR20] Krist AH, Davidson KW, Mangione CM, Barry MJ, Cabana M, Caughey AB et al (2021) Screening for Lung Cancer: US Preventive Services Task Force Recommendation Statement. JAMA 325:962–970. 10.1001/jama.2021.1117 33687470 10.1001/jama.2021.1117

[CR21] Krokstad S, Langhammer A, Hveem K, Holmen TL, Midthjell K, Stene TR et al (2013) Cohort Profile: the HUNT Study, Norway. Int J Epidemiol 42:968–977. 10.1093/ije/dys095 22879362 10.1093/ije/dys095

[CR22] Li H, Yang L, Zhao X, Wang J, Qian J, Chen H et al (2012) Prediction of lung cancer risk in a Chinese population using a multifactorial genetic model. BMC Med Genet 13:118. 10.1186/1471-2350-13-118 23228068 10.1186/1471-2350-13-118 PMC3573944

[CR23] Long E, Patel H, Byun J, Amos CI, Choi J (2022) Functional studies of lung cancer GWAS beyond association. Hum Mol Genet 31:R22–r36. 10.1093/hmg/ddac140 35776125 10.1093/hmg/ddac140 PMC9585683

[CR24] Magnussen JA, Morten; Granaas T, Magelssen M, Syse A, Celius EG, Klovning A (2015) Syversen, Iselin Dahlen. På ramme alvor Alvorlighet og prioritering. pp. 48

[CR25] Marcus MW, Raji OY, Duffy SW, Young RP, Hopkins RJ, Field JK (2016) Incorporating epistasis interaction of genetic susceptibility single nucleotide polymorphisms in a lung cancer risk prediction model. Int J Oncol 49:361–370. 10.3892/ijo.2016.3499 27121382 10.3892/ijo.2016.3499 PMC4902078

[CR26] Markaki M, Tsamardinos I, Langhammer A, Lagani V, Hveem K, Roe OD (2018) A validated clinical risk prediction model for lung Cancer in smokers of all ages and exposure types: a HUNT study. EBioMedicine 31:36–46. 10.1016/j.ebiom.2018.03.027 29678673 10.1016/j.ebiom.2018.03.027 PMC6013755

[CR27] McKay JD, Hung RJ, Gaborieau V, Boffetta P, Chabrier A, Byrnes G et al (2008) Lung cancer susceptibility locus at 5p15.33. Nat Genet 40:1404–1406. 10.1038/ng.254 18978790 10.1038/ng.254 PMC2748187

[CR28] McKay JD, Hung RJ, Han Y, Zong X, Carreras-Torres R, Christiani DC et al (2017) Large-scale association analysis identifies new lung cancer susceptibility loci and heterogeneity in genetic susceptibility across histological subtypes. Nat Genet 49:1126–1132. 10.1038/ng.3892 28604730 10.1038/ng.3892 PMC5510465

[CR29] Nguyen OTD, Fotopoulos I, Markaki M, Tsamardinos I, Lagani V, Røe OD (2024) Improving lung cancer screening selection: the HUNT Lung Cancer Risk Model for ever-smokers versus the NELSON and 2021 USPSTF criteria in the cohort of Norway (CONOR), a Population-based prospective study. JTO Clinical and. 100660. 10.1016/j.jtocrr.2024.100660. Research Reports 10.1016/j.jtocrr.2024.100660 PMC10998221 38586302

[CR30] Norheim O, Allgott B, Gjul G, Kjellevold A, Moen A, Sjøli S et al (2014) NOU Åpent og rettferdig – prioriteringer i helsetjenesten. pp. 128

[CR31] Norway, CRo (2023) Årsrapport 2022 med resultater og forbedringstiltak fra nasjonalt kvalitetsregister for lungekreft. Cancer Registry nor. https://www.kreftregisteret.no/globalassets/publikasjoner-og-rapporter/arsrapporter/publisert-2023/arsrapport-2022-nasjonalt-kvalitetsregister-for-lungekreft.pdf. Accessed 13 Aug 2023

[CR32] HUNT Fast Track GWAS catalogue. NTNU/HUNT. https://www.ntnu.edu/hunt/data. Accessed 25 Oct 2023

[CR11] Office for Health Improvement and Disparities (2020) Guidance cost utility analysis: health economic studies. Government of United Kingdom. https://www.gov.uk/guidance/cost-utility-analysis-health-economic-studies. Accessed 27 Aug 2023

[CR33] Ottersen T, Førde R, Kakad M, Kjellevold A, Melberg HO, Moen A et al (2016) A new proposal for priority setting in Norway: open and fair. Health Policy 120:246–251. 10.1016/j.healthpol.2016.01.012 26851991 10.1016/j.healthpol.2016.01.012

[CR34] Pinsky PF, Berg CD (2012) Applying the National Lung Screening Trial eligibility criteria to the US population: what percent of the population and of incident lung cancers would be covered? J Med Screen 19:154–156. 10.1258/jms.2012.012010 23060474 10.1258/jms.2012.012010

[CR35] Qian DC, Han Y, Byun J, Shin HR, Hung RJ, McLaughlin JR et al (2016) A novel pathway-based Approach improves Lung Cancer Risk Prediction using germline genetic variations. Cancer epidemiology, biomarkers & prevention: a publication of the American Association for Cancer Research, cosponsored by the American Society of Preventive Oncology. 25:1208–1215. 10.1158/1055-9965.Epi-15-1318 10.1158/1055-9965.EPI-15-1318 PMC5492390 27222311

[CR36] Raji OY, Agbaje OF, Duffy SW, Cassidy A, Field JK (2010) Incorporation of a genetic factor into an epidemiologic model for prediction of individual risk of lung cancer: the Liverpool Lung Project. Cancer prevention research (Philadelphia. Pa) 3:664–669. 10.1158/1940-6207.Capr-09-0141 10.1158/1940-6207.CAPR-09-0141 20424129

[CR38] Røe OD, Markaki M, Tsamardinos I, Lagani V, Nguyen OTD, Pedersen JH et al (2019) Reduced’ HUNT model outperforms NLST and NELSON study criteria in predicting lung cancer in the Danish screening trial. BMJ Open Respir Res 6:e000512. 10.1136/bmjresp-2019-000512 31803478 10.1136/bmjresp-2019-000512 PMC6890385

[CR37] Royston P, Altman DG (2013) External validation of a Cox prognostic model: principles and methods. BMC Med Res Methodol 13:33. 10.1186/1471-2288-13-33 23496923 10.1186/1471-2288-13-33 PMC3667097

[CR39] Spitz MR, Amos CI, Land S, Wu X, Dong Q, Wenzlaff AS et al (2013) Role of selected genetic variants in lung cancer risk in African americans. J Thorac Oncol 8:391–397. 10.1097/JTO.0b013e318283da29 23454887 10.1097/JTO.0b013e318283da29 PMC3623962

[CR41] Steyerberg EW, Vergouwe Y (2014) Towards better clinical prediction models: seven steps for development and an ABCD for validation. Eur Heart J 35:1925–1931. 10.1093/eurheartj/ehu207 24898551 10.1093/eurheartj/ehu207 PMC4155437

[CR40] Steyerberg EW, Eijkemans MJC, Habbema JDF (2001) Application of shrinkage techniques in logistic regression analysis: a Case Study. Stat Neerl 55:76–88. 10.1111/1467-9574.00157

[CR42] Tammemägi MC, Ruparel M, Tremblay A, Myers R, Mayo J, Yee J et al (2022) USPSTF2013 versus PLCOm2012 lung cancer screening eligibility criteria (International Lung Screening Trial): interim analysis of a prospective cohort study. Lancet Oncol 23:138–148. 10.1016/s1470-2045(21)00590-8 34902336 10.1016/S1470-2045(21)00590-8 PMC8716337

[CR43] Timofeeva M, Kropp S, Sauter W, Beckmann L, Rosenberger A, Illig T et al (2010) Genetic polymorphisms of MPO, GSTT1, GSTM1, GSTP1, EPHX1 and NQO1 as risk factors of early-onset lung cancer. Int J Cancer 127:1547–1561. 10.1002/ijc.25175 20091863 10.1002/ijc.25175

[CR44] Ubel PA, Hirth RA, Chernew ME, Fendrick AM (2003) What is the price of life and why doesn’t it increase at the rate of inflation? Arch Intern Med 163:1637–1641. 10.1001/archinte.163.14.1637 12885677 10.1001/archinte.163.14.1637

[CR45] Weissfeld JL, Lin Y, Lin HM, Kurland BF, Wilson DO, Fuhrman CR et al (2015) Lung Cancer Risk Prediction using common SNPs located in GWAS-Identified susceptibility regions. J Thorac Oncol 10:1538–1545. 10.1097/jto.0000000000000666 26352532 10.1097/JTO.0000000000000666 PMC4636453

[CR46] Wetterstrand KA, National Human Genome Research Institute (2021) The Cost of Sequencing a Human Genome. https://www.genome.gov/about-genomics/fact-sheets/Sequencing-Human-Genome-cost. Accessed 18 Sept 2023

[CR47] Young RP, Hopkins RJ, Hay BA, Epton MJ, Mills GD, Black PN et al (2009) A gene-based risk score for lung cancer susceptibility in smokers and ex-smokers. Postgrad Med J 85:515–524. 10.1136/pgmj.2008.077107 19789190 10.1136/pgmj.2008.077107

