# Supplementary material for: The HUNT lung-SNP model: genetic variants plus clinical variables improve lung cancer risk assessment over clinical models
Source: J Cancer Res Clin Oncol. 2024 Aug 12;150(8):389. doi: 10.1007/s00432-024-05909-w (PMC11317451; doi:10.1007/s00432-024-05909-w)
Supplement: Supplementary file 1 — Supplementary Material 1 [file 432_2024_5909_MOESM1_ESM.docx]

**The HUNT Lung-SNP model: genetic variants plus clinical variables improve lung cancer risk assessment over clinical models.**

**Olav Toai Duc Nguyen, Ioannis Fotopoulos, Therese Haugdahl Nøst, Maria Markaki, Ioannis Tsamardinos, Vincenzo Lagani, Oluf Dimitri Røe**

**Supplementary Materials**

**Table of Contents**

| **Supplementary Methods**  - Validation dataset  - Genotyping  - Univariate analysis  - Multivariable modeling  - Statistical Testing  - The NLST, NELSON and 2021 USPSTF criteria | **Page 3-4** |
| --- | --- |
| **Supplementary Results** | **Page 5-6** |
| **Supplementary Table 1** | **Page 7** |
| **Supplementary Table 2** | **Page 8-9** |
| **Supplementary Table 3** | **Page 10** |
| **Supplementary Table 4** | **Page 11** |
| **Supplementary Table 5** | **Page 12** |
| **Supplementary Table 6** | **Page 13** |
| **Supplementary Table 7** | **Page 14** |
| **Supplementary Table 8** | **Page 15** |
| **Supplementary Table 9** | **Page 16** |
| **Supplementary Table 10** | **Page 17** |
| **Supplementary Table 11** | **Page 18** |
| **Supplementary Fig. 1** | **Page 19** |
| **Supplementary Fig. 2** | **Page 20** |
| **Supplementary Fig. 3** | **Page 21** |
| **Supplementary Fig. 4** | **Page 22** |
| **Supplementary Fig. 5** | **Page 23** |
| **Supplementary Fig. 6** | **Page 24** |

**Supplementary Methods**

**Validation dataset**

The Tromsø Study was conducted in the municipality of Tromsø in North-Norway. Tromsø 4 was conducted in 1994-1995 and included 27158 persons aged 25-97 years, Tromsø 5 was conducted in 2001-2002 and included 8130 persons aged 30-89 years and Tromsø 6 was conducted in 2007-2008 and included 12984 persons aged 30-87 years.

A total of 6572 individuals from the fourth, fifth and sixth survey of the Tromsø Study (Tromsø 4 (n=5293), 5 (n=48) and 6 (n=1231)) were genotyped.

**Genotyping**

The DNA from the HUNT2 samples was genotyped using one of three different Illumina Human Core Exome arrays: HumanCoreExome12 v1.0, HumanCoreExome12 v1.1 and UM HUNT Biobank v1.0. All genotyping was performed at the Genomics‐Core Facility (GCF) at the Norwegian University of Science and Technology, NTNU. The HUNT2 single nucleotide polymorphism (SNP) dataset has been produced and quality controlled at the K.G. Jebsen Center for Genetic Epidemiology, NTNU, in collaboration with the University of Michigan, USA. All missing values in the SNPs have been imputed. The imputation and quality control of the datasets were done as following: Minimac3 (v2.0.1, http://genome.sph.umich.edu/wiki/Minimac3^)1^ was used in the performance of imputation, with default settings (2.5 Mb reference based chunking with 500kb windows) and a customized Haplotype Reference consortium release 1.1 (HRC v1.1) for autosomal variants and HRC v1.1 for chromosome X variants.^2^ The customized reference panel represented the merged panel of two reciprocally imputed reference panels: (1) 2,201 low‐coverage whole‐genome sequences samples from the HUNT study and (2) HRC v1.1 with 1,023 HUNT WGS samples removed before merging. Imputed variants with Rsq < 0.3 were excluded.^3^

The lung cancer (LC) associated SNPs were selected manually from the HUNT Fast-track catalog^4^ where all SNPs have a verified high level of association with LC in published literature (P < 5x10^‐ 8^, Supplementary Table 1). The key words lung cancer, adenocarcinoma, squamous cell carcinoma and small cell carcinoma were used to identify SNPs within the catalog.

**Univariate analysis**

The SNP genotypes were transformed into ordinal encodings as described in the literature.^5^ Specifically, each SNP was coded as zero when the minor allele is homozygous (-/-), one for heterozygous (+/-), and two for major allele homozygous (+/+) genotypes.

**Multivariable modeling**

Model training was performed as follows: The outcome was defined as the truth value of “diagnosis of LC within 6 years”, leading to a binary classification task. The model for assessing LC risk was fit using the original eight HUNT LCM clinical covariates along with the 22 SNP genotype predictors. The SNP genotypes were transformed into ordinal encodings as described above.

To fit the final model, 100 logistic regression models were fitted on 100 bootstrap resamples (i.e., resampling with replacement). At each iteration, a resampled dataset is selected with replacement from the original sample, and a logistic regression model is fitted on the resampled dataset. A shrinkage coefficient is then estimated as the percentage of variance explained with respect to the logistic regression model fitted on the original dataset.^6, 7^ The final HUNT Lung-SNP model is obtained by (i) averaging the 100 shrinkage coefficients, and (ii) multiplying this average by the coefficients of the logistic regression model fitted on the original dataset.^8^ This resampling strategy reduces the probability of overfitting by addressing the effect of class imbalance.

**Model validation and the top 16^th^ percentile risk threshold**

To stratify individuals in high- and low-risk categories according to the HUNT Lung-SNP and HUNT LCM risk scores, a cut-off for each model was derived corresponding to the top 16th percentile of their respective in-sample predictions. This cutpoint was used to equal the risk threshold of our original paper of the HUNT LCM.^9^ It is not optimizing any trade-off in our data according to some selective results, but used based upon the work by Royston et al.^10^, which states: “For a given number of groups, the method is designed to minimise the loss of information that occurs with grouping. The required cut-points are the 16th, 50th and 84th centiles of the continuous variable, here the prognostic index in the derivation dataset. On a standard Normal scale, these correspond to 0 and approximately ±1, i.e., mean ± 1SD.”

**Statistical Testing**

We use a permutation approach for assessing the statistical significance of differences in sensitivity, specificity, positive predictive value (PPV) and negative predictive value (NPV). For any two models, namely m0 and m1, let p0i be the prediction for subject i provided by model m0 and p1i be the prediction provided by m1 for the same subject. For each subject i we swap p0i and p1i with a 50% probability, and then we calculate the four relevant metrics. We repeat this process 10000 times to estimate the null distribution of the differences in sensitivity, specificity, PPV and NPV. Statistical significance is computed by contrasting the original differences against their respective empirical null distribution (two-tailed test).

**The NLST, NELSON and 2021 USPSTF criteria**

The clinical set of criteria used in this study are defined as follows:

NLST: age between 55 and 74 years old, at least 30 pack-years, at most 15 years quit time.

NELSON: age between 50 to 74 years old, >15 cigarettes per day for >25 years or >10 cigarettes per day for >30 years, quit smoking ≤10 years.

2021 USPSTF: age between 50 to 80 years old, at least 20 pack-years, and currently smoking or quit smoking <15 years.

**Supplementary Results**

**Contrasting the HUNT Lung-SNP and HUNT LCM model against the NLST, NELSON and 2021 USPSTF criteria**

In the HUNT2 cohort, the application of the NLST, NELSON and 2021 USPSTF criteria led to the identification of 1233, 2324, 4010 subjects at high risk, respectively. The number of detected LC in six years in each selection was 31, 44 and 78, corresponding to a sensitivity of 19.38%, 27.50% and 48.75%, respectively (Supplementary Table 7, 8 and 9). When the same numbers of high-risk individuals were selected according to the HUNT Lung-SNP model, the number of detected LC amounted to 52, 76 and 103, and corresponding sensitivities to 32.50%, 47.50% and 64.38% respectively. Based on^9^, the maximum of these three p-values (which is<0.01) is a valid criterion for assessing whether the conjunction of the three null hypotheses can be all rejected, thus adjusting for multiple testing. The same was found when the same numbers of high-risk individuals were selected according to the HUNT LCM, where the number of detected LC were 41, 70, 91 and the corresponding sensitivities 25.62% (p<0.05), 43.75% (p<0.01) and 56.88% (p<0.05) respectively (Supplementary Tables 7, 8 and 9).

In terms of NNS to identify one LC case, the HUNT Lung-SNP was the most well-performing model, with NNS of 24 vs. 40 (NLST), 31 vs. 53 (NELSON) and 39 vs. 51 (USPSTF), p<0.01 for all comparisons (Supplementary Fig. 2A-C). The HUNT LCM model had also lower NNS than the clinical criteria in the HUNT2 cohort, with NNS of 30 vs. 40 (NLST, p>0.05), 33 vs. 53 (NELSON, p<0.01) and 44 vs. 51 (USPSTF, p<0.05).

By applying the top 16th percentile as a cutoff for risk stratification the HUNT Lung-SNP identified about 280%, 168% and 50% more cases in the HUNT2 ever-smokers in six years compared to the NLST, NELSON and USPSTF criteria respectively (Supplementary Fig. 1). This improvement leads to a significantly reduced number of screenings needed per cancer detected for the HUNT Lung-SNP compared to several of the criteria comparisons (Supplementary Fig. 2A-C). These results show that the HUNT Lung-SNP represented the most effective of these models for screening the same number of individuals.

In the Tromsø validation cohort, the HUNT Lung-SNP model outperformed the NELSON and 2021 USPSTF criteria in terms of sensitivity (61.54% vs. 25.64% and 74.36% vs. 43.59%, respectively), PPV (11.65% vs. 4.85% and 9.27% vs. 5.43%, respectively) and NPV (99.39% vs. 98.82% and 99.57% vs. 99.06%, respectively) (p<0.01) (Supplementary Table 8 and 9). The HUNT Lung-SNP model achieved also better sensitivity (38.46% vs. 23.08%), specificity (96.72% vs. 96.49%), PPV (14.85% vs. 8.91%) and NPV (99.06% vs. 98.83%) against the NLST criteria, but none of these results reached statistical significance. Furthermore, the HUNT Lung-SNP had lower NNS than any set of clinical criteria, with statistically significant differences against the NELSON (9 vs. 21) and 2021 USPSTF criteria (11 vs. 18), but not against the NLST (7 vs. 11) (Supplementary Fig. 3A-C).

The same analysis was performed with HUNT LCM in the Tromsø cohort with similar results, the HUNT LCM model outperformed the NELSON and 2021 USPSTF criteria, in terms of sensitivity ( 56.41% vs. 25.64% and 58.97% vs. 43.59%, respectively) (p<0.01), PPV (10.68% vs. 4.85% (p<0.01) and 7.35% vs. 5.43% (p<0.05), respectively) and NPV (99.31% vs. 98.82% and 99.32% vs. 99.06%, respectively) (p<0.01) (Supplementary Table 8 and 9). The HUNT LCM also achieved significantly better sensitivity (48.72% vs. 23.08%, p<0.01) and NPV (99.22% vs. 98.83%, p<0.01) compared to the NLST criteria. Finally, the HUNT LCM had significantly lower NNS than any set of clinical criteria: 5 vs. 11 (NLST), 9 vs. 21 (NELSON) and 14 vs. 18 (USPSTF) (Supplementary Fig. 3A-C).

**Cost of SNP analysis**

The administrative costs related to blood drawing in our public hospital is estimated to $14 USD per blood test, while the cost of the genetic analysis of the SNP-panel including all the 22 SNPs can be estimated to $23 USD per analysis,^10^ resulting in a total of $37. Based on this price, the additional cost will be $1.137.713 USD (=$37USD/individuals x 30749 individuals). When using the top 16^th^ percentile, the HUNT Lung-SNP predicted 21 unique cases in six years (Supplementary Table 9) and the HUNT LCM predicted 6 unique cases, thus 15 surplus LC cases were predicted by the SNP model. Based on previous publications,^11, 12^ an average of YLL per LC case is estimated to be 15 years, given survival from LC (i.e., a total of 225 YLL for these 15 cases), resulting in a cost per YLL saved of $5056 USD given that all the 15 cases survive from lung cancer. However, it is unlikely that all 15 individuals will survive from lung cancer. Based on a 5-year relative survival rate of 68% for patients with lung cancer stage I,^13^ it is probably more realistic to calculate an average YLL per lung cancer case of 10 years, which will result in a cost per YLL saved of $7584 USD.

YLL cannot translate directly to quality-adjusted life years (QALY). A health-related quality of life (HRQL) score of 0.75^14^ was applied, meaning that 3/4 of the time saved represents life in full health (=10 YLL x 0.75 = 7.5 QALY), resulting in a cost of $10.113 USD/QALY for the SNP-analysis.

**Supplementary Table 1.** Descriptive statistics for the 22 SNP measured on the HUNT2 participants that ever smoked. For each SNP we report the rs ID, gene name, known association with lung cancer(s), occurrence by ethnicity, allele frequency, and statistical association with lung cancer diagnosis at six years.

​​

| **SNP ID (rs)** | **Gene symbol** | **Association** | **Alleles** | **Ethnicity** | **Controls**  **n=30589** | **Cases**  **n=160** | **P-value*** |
| --- | --- | --- | --- | --- | --- | --- | --- |
| **1051730** | CHRNA3 | nicotine dependence, lung carcinoma,  lung adenocarcinoma, smoking behaviour | 1 | Asian^15^, Caucasian^16, 17^, Latin American^18^, African American^19^ | 45 % | 53 % | 0.005 |
|  |  |  | 2 |  | 11 % | 15 % |  |
| **8042374** | CHRNA3 | lung carcinoma | 1 | Caucasian^20^, Asian^21^ | 34 % | 28 % | 0.019 |
|  |  |  | 2 |  | 5 % | 3 % |  |
| **8034191** | HYKK | lung carcinoma | 1 | Caucasian^22^ | 45 % | 53 % | 0.023 |
|  |  |  | 2 |  | 12 % | 14 % |  |
| **13314271** | TP63 | lung adenocarcinoma | 1 | Caucasian^23^ | 49 % | 54 % | 0.041 |
|  |  |  | 2 |  | 30 % | 22 % |  |
| **4488809** | TP63 | lung carcinoma | 1 | Asian^24^ | 49 % | 54 % | 0.041 |
|  |  |  | 2 |  | 30 % | 22 % |  |
| **11571833** | BRCA2 | lung carcinoma, squamous cell carcinoma, small cell lung cancer | 1 | Caucasian^23^ | 1 % | 3 % | 0.042 |
|  |  |  | 2 |  | 0 % | 0 % |  |
| **10937405** | TP63 | lung adenocarcinoma | 1 | Asian^25^, Caucasian^26^ | 49 % | 48 % | 0.096 |
|  |  |  | 2 |  | 24 % | 19 % |  |
| **2494938** | LRFN2 | lung carcinoma, squamous cell carcinoma | 1 | Asian^27^ | 50 % | 49 % | 0.235 |
|  |  |  | 2 |  | 28 % | 31 % |  |
| **31489** | CLPTM1L | lung adenocarcinoma | 1 | Caucasian^16^, Asian^28^ | 48 % | 51 % | 0.337 |
|  |  |  | 2 |  | 16 % | 12 % |  |
| **401681** | CLPTM1L | lung carcinoma | 1 | Caucasian^20, 29^ | 49 % | 55 % | 0.450 |
|  |  |  | 2 |  | 19 % | 14 % |  |
| **2131877** | XXYLT1 | non-small cell lung carcinoma | 1 | Asian^30^ | 30 % | 34 % | 0.455 |
|  |  |  | 2 |  | 3 % | 3 % |  |
| **4975616** | MIR4457, CLPTM1L | lung carcinoma | 1 | Caucasian^31^ | 48 % | 53 % | 0.471 |
|  |  |  | 2 |  | 34 % | 34 % |  |
| **12296850** | NR1H4,  SLC17A8 | non-small cell lung carcinoma | 1 | Asian^32^ | 11 % | 12 % | 0.501 |
|  |  |  | 2 |  | 0 % | 1 % |  |
| **17879961** | CHEK2 | lung carcinoma, squamous cell carcinoma | 1 | Caucasian^23^ | 1 % | 1 % | 0.624 |
|  |  |  | 2 |  | 0 % | 0 % |  |
| **753955** | LOC105370113 | lung carcinoma | 1 | Asian^24^ | 46 % | 43 % | 0.655 |
|  |  |  | 2 |  | 41 % | 43 % |  |
| **2285947** | DNAH11 | lung carcinoma, squamous cell carcinoma | 1 | Asian^27^ | 50 % | 50 % | 0.660 |
|  |  |  | 2 |  | 26 % | 27 % |  |
| **7216064** | BPTF | lung adenocarcinoma, lung carcinoma | 1 | Asian^33^ | 33 % | 30 % | 0.689 |
|  |  |  | 2 |  | 4 % | 6 % |  |
| **36600** | MTMR3 | lung carcinoma | 1 | Asian^24^ | 36 % | 35 % | 0.721 |
|  |  |  | 2 |  | 58 % | 59 % |  |
| **2736100** | TERT | lung adenocarcinoma, lung carcinoma | 1 | Asian^25^, Caucasian^34^, African American^35^ | 50 % | 46 % | 0.755 |
|  |  |  | 2 |  | 25 % | 26 % |  |
| **9387478** | DCBLD1 | lung carcinoma | 1 | Asian^36^ | 50 % | 54 % | 0.760 |
|  |  |  | 2 |  | 26 % | 23 % |  |
| **2853677** | TERT | lung adenocarcinoma | 1 | Caucasian^37^, Asian^33^, African American^38^ | 50 % | 49 % | 0.826 |
|  |  |  | 2 |  | 32 % | 33 % |  |
| **7086803** | VTI1A | lung carcinoma | 1 | Asian^36^ | 4 % | 4 % | 0.963 |
|  |  |  | 2 |  | 0 % | 0 % |  |

*Proportional odds likelihood ratio test

**Supplementary Table 2.** Descriptive statistics for the 22 SNP measured on the Tromsø participants that ever smoked. For each SNP we report the rs ID, gene name, allele frequency, and statistical association with lung cancer diagnosis at six years. Known association with lung cancer(s) and occurrence by ethnicity, see Supplementary Table 1.

| **SNP ID (rs)** | **Gene symbol** | **Alleles** | **Controls**  **n=2624** | **Cases**  **n=39** | **P-value*** |
| --- | --- | --- | --- | --- | --- |
| **13314271** | TP63 | 0 | 21.6 % | 46.2 % | 0.001 |
|  |  | 1 | 51.0 % | 33.3 % |  |
|  |  | 2 | 27.5 % | 20.5 % |  |
| **4488809** | TP63 | 0 | 21.6 % | 46.2 % | 0.001 |
|  |  | 1 | 50.9 % | 33.3 % |  |
|  |  | 2 | 27.5 % | 20.5 % |  |
| **10937405** | TP63 | 0 | 27.9 % | 51.3 % | 0.003 |
|  |  | 1 | 49.7 % | 41.0 % |  |
|  |  | 2 | 22.4 % | 7.7 % |  |
| **8042374** | CHRNA3 | 0 | 54.7 % | 66.7 % | 0.151 |
|  |  | 1 | 38.9 % | 33.3 % |  |
|  |  | 2 | 6.3 % | 0 % |  |
| **1051730** | CHRNA3 | 0 | 50.9 % | 35.9 % | 0.174 |
|  |  | 1 | 40.1 % | 51.3 % |  |
|  |  | 2 | 9.1 % | 12.8 % |  |
| **8034191** | HYKK | 0 | 50.2 % | 35.9 % | 0.206 |
|  |  | 1 | 40.3 % | 51.3 % |  |
|  |  | 2 | 9.5 % | 12.8 % |  |
| **753955** | LOC105370113 | 0 | 14.4 % | 7.7 % | 0.301 |
|  |  | 1 | 45.5 % | 56.4 % |  |
|  |  | 2 | 40.1 % | 35.9 % |  |
| **2736100** | TERT | 0 | 30.9 % | 41.0 % | 0.353 |
|  |  | 1 | 44.5 % | 41.0 % |  |
|  |  | 2 | 24.7 % | 17.9 % |  |
| **2285947** | DNAH11 | 0 | 25.8 % | 30.8 % | 0.318 |
|  |  | 1 | 50.5 % | 38.5 % |  |
|  |  | 2 | 23.7 % | 30.8 % |  |
| **401681** | CLPTM1L | 0 | 30.7 % | 35.9 % | 0.513 |
|  |  | 1 | 49.4 % | 51.3 % |  |
|  |  | 2 | 19.9 % | 12.8 % |  |
| **2494938** | LRFN2 | 0 | 22.5 % | 28.2 % | 0.522 |
|  |  | 1 | 49.7 % | 51.3 % |  |
|  |  | 2 | 27.8 % | 20.5 % |  |
| **17879961** | CHEK2 | 0 | 99.0 % | 100.0 % | 0.540 |
|  |  | 1 | 1.0 % | 0 % |  |
|  |  | 2 | 0 % | 0 % |  |
| **31489** | CLPTM1L | 0 | 35.6 % | 38.5 % | 0.608 |
|  |  | 1 | 48.3 % | 51.3 % |  |
|  |  | 2 | 16.2 % | 10.3 % |  |
| **4975616** | MIR4457, CLPTM1L | 0 | 18.8 % | 12.8 % | 0.615 |
|  |  | 1 | 48.5 % | 53.8 % |  |
|  |  | 2 | 32.7 % | 33.3 % |  |
| **2853677** | TERT | 0 | 20.9 % | 23.1 % | 0.667 |
|  |  | 1 | 44.0 % | 48.7 % |  |
|  |  | 2 | 35.1 % | 28.2 % |  |
| **11571833** | BRCA2 | 0 | 98.3 % | 100.0 % | 0.717 |
|  |  | 1 | 1.6 % | 0 % |  |
|  |  | 2 | 0 % | 0 % |  |
| **12296850** | NR1H4, SLC17A8 | 0 | 88.1 % | 84.6 % | 0.747 |
|  |  | 1 | 11.7 % | 15.4 % |  |
|  |  | 2 | 0.2 % | 0 % |  |
| **7086803** | VTI1A | 0 | 96.9 % | 97.4 % | 0.841 |
|  |  | 1 | 3.1 % | 2.6 % |  |
|  |  | 2 | 0 % | 0 % |  |
| **7216064** | BPTF | 0 | 66.0 % | 66.7 % | 0.875 |
|  |  | 1 | 30.3 % | 28.2 % |  |
|  |  | 2 | 3.7 % | 5.1 % |  |
| **9387478** | DCBLD1 | 0 | 26.0 % | 23.1 % | 0.879 |
|  |  | 1 | 48.8 % | 48.7 % |  |
|  |  | 2 | 25.2 % | 28.2 % |  |
| **2131877** | XXYLT1 | 0 | 72.1 % | 74.4 % | 0.946 |
|  |  | 1 | 24.8 % | 23.1 % |  |
|  |  | 2 | 3.1 % | 2.6 % |  |
| **36600** | MTMR3 | 0 | 6.7 % | 7.7 % | 0.968 |
|  |  | 1 | 39.7 % | 38.5 % |  |
|  |  | 2 | 53.6 % | 53.8 % |  |

*Proportional odds likelihood ratio test

**Supplementary Table 3.** Logistic regression model coefficients for the HUNT LCM and HUNT-SNP models in the HUNT2 population.

|  | **HUNT LCM** | **22 SNPs** | **HUNT Lung-SNP** |
| --- | --- | --- | --- |
| **AUC [95% CI]** | **0.844 [0.820-0.869]** | **0.625 [0.583-0.666]** | **0.875 [0.854-0.896]** |
| **Intercept** | **1.1820** | **-2.41455653696287** | **4.21778580654473** |
| **Sex** | **0.3157** |  | 0.190020458738857 |
| **Age (100/Age)**  (standard error; standardized coefficient) | **-1.9850**  (0.7307; -2.7166) |  | -2.67509996729452 |
| **PackYrs (log)**  (standard error; standardized coefficient) | **1.1199**  (0.8824; 1.2691) |  | 0.237568515138304 |
| **CougDy** | **0.4921** |  | 0.537266783481621 |
| **SmoExpH (log)**  (standard error; standardized coefficient) | **0.0807**  (0.9037; 0.0893) |  | -0.0181932859869948 |
| **Quit time (log)**  (standard error; standardized coefficient) | **-0.2402**  (1.3540; -0.1774) |  | -0.387467807235083 |
| SmoCigDyN (standard error; standardized coefficient) | **-0.0400** (6.7172; -0.0059) |  | 0.0119423766824795 |
| **BMI (log)**  (standard error; standardized coefficient) | **-1.7024**  (0.1429; -11.9130) |  | -1.52797647758964 |
| **SNPs ID (rs)** | **Alleles** |  |  |
| **rs1051730** | 1 | 0.301335993595137 | 0.599157420666742 |
|  | 2 | 0.646906363834462 | 1.26808436357984 |
| **rs11571833 :** | 1 | 0.403721095369583 | 0.902779752263699 |
| **rs13314271 :** | 1 | 1.53496446386189 | 2.5670396373813 |
|  | 2 | 3.31322048832307 | 5.08958813709906 |
| **rs4488809 :** | 1 | -1.53184129693468 | -2.54530083652787 |
|  | 2 | -3.48243122835638 | -5.40504837573278 |
| **rs9387478 :** | 1 | 0.0596458100450334 | 0.141767211090145 |
|  | 2 | -0.0291343897762805 | -0.042894460504954 |
| **rs10937405 :** | 1 | -0.0677262870609957 | -0.195829092952348 |
|  | 2 | -0.0439777594587069 | -0.133072209051457 |
| **rs12296850 :** | 1 | 0.403721095369583 | 0.0777808955681538 |
|  | 2 | 0.358692625512479 | 0.823292942032411 |
| **rs17879961 :** | 1 | -0.21085899120742 | -0.358517087283351 |
|  | 2 | -2.12283639098253 | -6.40576696198426 |
| **rs2131877 :** | 1 | 0.0907912301044699 | 0.174139039056495 |
|  | 2 | -0.0923436048099337 | -0.206688987198503 |
| **rs2285947 :** | 1 | 0.0308954774662489 | 0.0579613042261956 |
|  | 2 | 0.0426772517280959 | 0.0759602025481811 |
| **rs2494938 :** | 1 | 0.0575332903254417 | 0.112138158048829 |
|  | 2 | 0.117557659628302 | 0.294519152991272 |
| **rs2736100 :** | 1 | -0.134873540639921 | -0.269042602916004 |
|  | 2 | -0.0742230141434793 | -0.121458982952246 |
| **rs2853677 :** | 1 | 0.0914344019094308 | 0.200046629685168 |
|  | 2 | 0.115048614491194 | 0.21122962622128 |
| **rs31489 :** | 1 | -0.138387215526567 | -0.349656071076508 |
|  | 2 | -0.151377862197269 | -0.345053063498084 |
| **rs36600 :** | 1 | 0.0121846782472839 | -0.00063409028772517 |
|  | 2 | 0.0364785436180288 | 0.0400456731610682 |
| **rs401681 :** | 1 | 0.138898043740575 | 0.327631537609551 |
|  | 2 | 0.0340680645458131 | 0.12432807569119 |
| **rs4975616 :** | 1 | 0.0687292022417741 | 0.187779041854502 |
|  | 2 | 0.0206935481251343 | 0.114718531788928 |
| **rs7086803 :** | 1 | -0.00431267844540745 | -0.00682996635156004 |
|  | 2 | -2.052980927752 | -3.8876576271527 |
| **rs7216064 :** | 1 | -0.0357589084105838 | -0.0596226602405666 |
|  | 2 | 0.191108886112439 | 0.421774768248072 |
| **rs753955 :** | 1 | -0.035489024340559 | -0.0367290643144561 |
|  | 2 | 0.0173706926373296 | 0.0634866554705837 |
| **rs8034191 :** | 1 | -0.138059983427769 | -0.304658411800255 |
|  | 2 | -0.492258023059165 | -0.998375043542052 |
| **rs8042374 :** | 1 | -0.100928422238912 | -0.18079715820545 |
|  | 2 | -0.214826841555479 | -0.456212514572205 |

**Supplementary Table 4.** Performance of the polygenic-clinical HUNT Lung-SNP model and the clinical HUNT Lung Cancer Model (HUNT LCM) in predicting lung cancer diagnosis within six years. The models were applied to the discovery cohort (HUNT2, n=30749), as well as on a validation cohort (Tromsø, n=2663). The top 16th percentile risk threshold for lung cancer risk computed on the HUNT2 cohort was used as a cutoff to separate high and low risk subjects.
CI, Confidence Interval; FN, false negative; FP, false positive; NPV, negative predictive value; PPV, positive predictive value; TN, true negative; TP, true positive.
^a^p>0.05, versus HUNT LCM
^b^p<0.05, versus HUNT LCM
^c^p<0.01, versus HUNT LCM

**
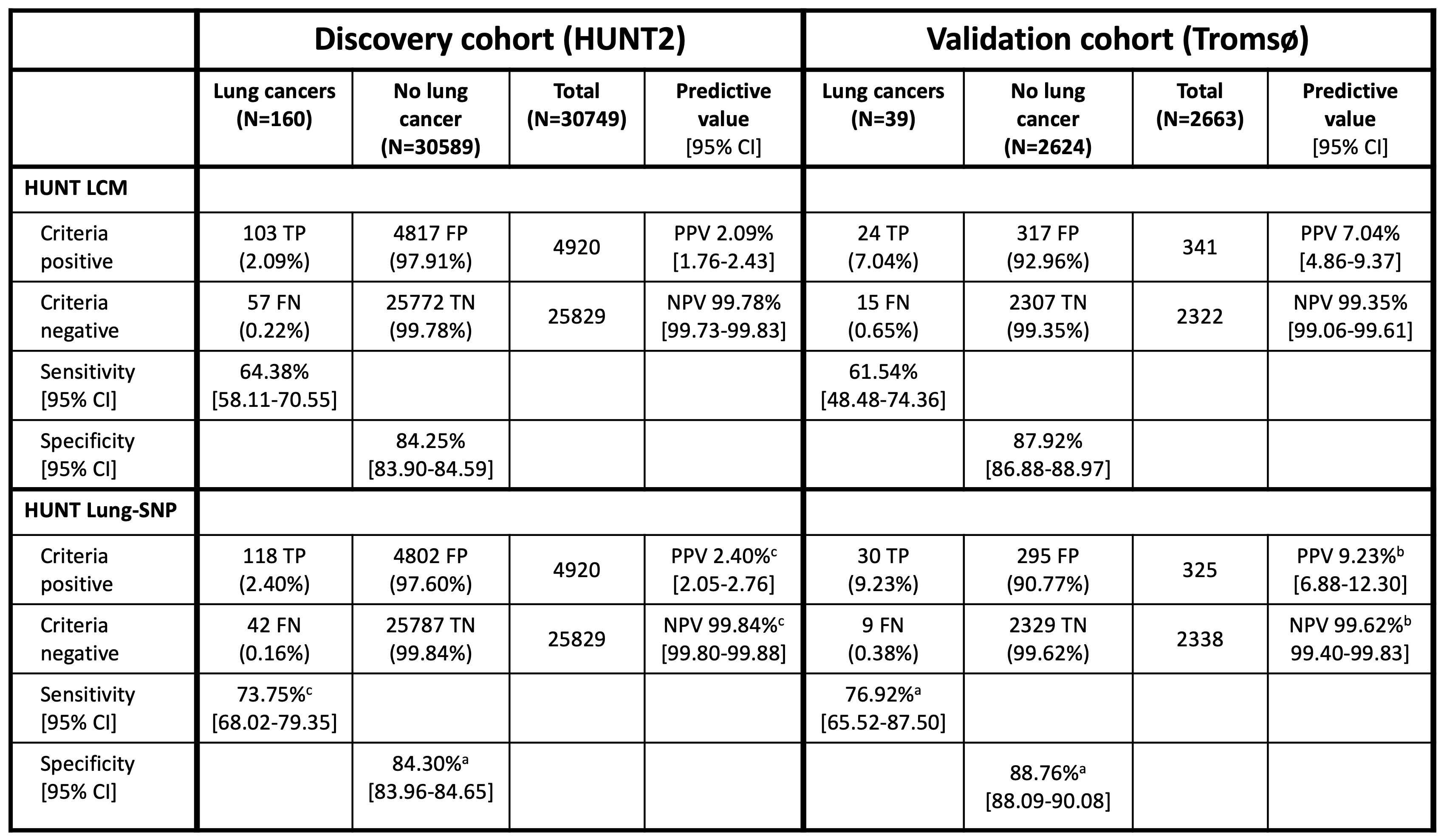
**

**Supplementary Table 5.** Performance of the polygenic-clinical HUNT Lung-SNP model and the clinical HUNT Lung Cancer Model (HUNT LCM) in predicting lung cancer diagnosis within six years. The models were applied to the HUNT2 subpopulations <60 years (n=21762) and ≥60 years (n=8987). The top 16th percentile risk threshold for lung cancer risk computed on the HUNT2 cohort was used as a cutoff to separate high and low risk subjects.
FN, false negative; FP, false positive; NPV, negative predictive value; PPV, positive predictive value; TN, true negative; TP, true positive; NNS, number of screenings needed per cancer detected.
^a^p>0.05, versus HUNT LCM
^b^p<0.05, versus HUNT LCM
^c^p<0.01, versus HUNT LCM

**
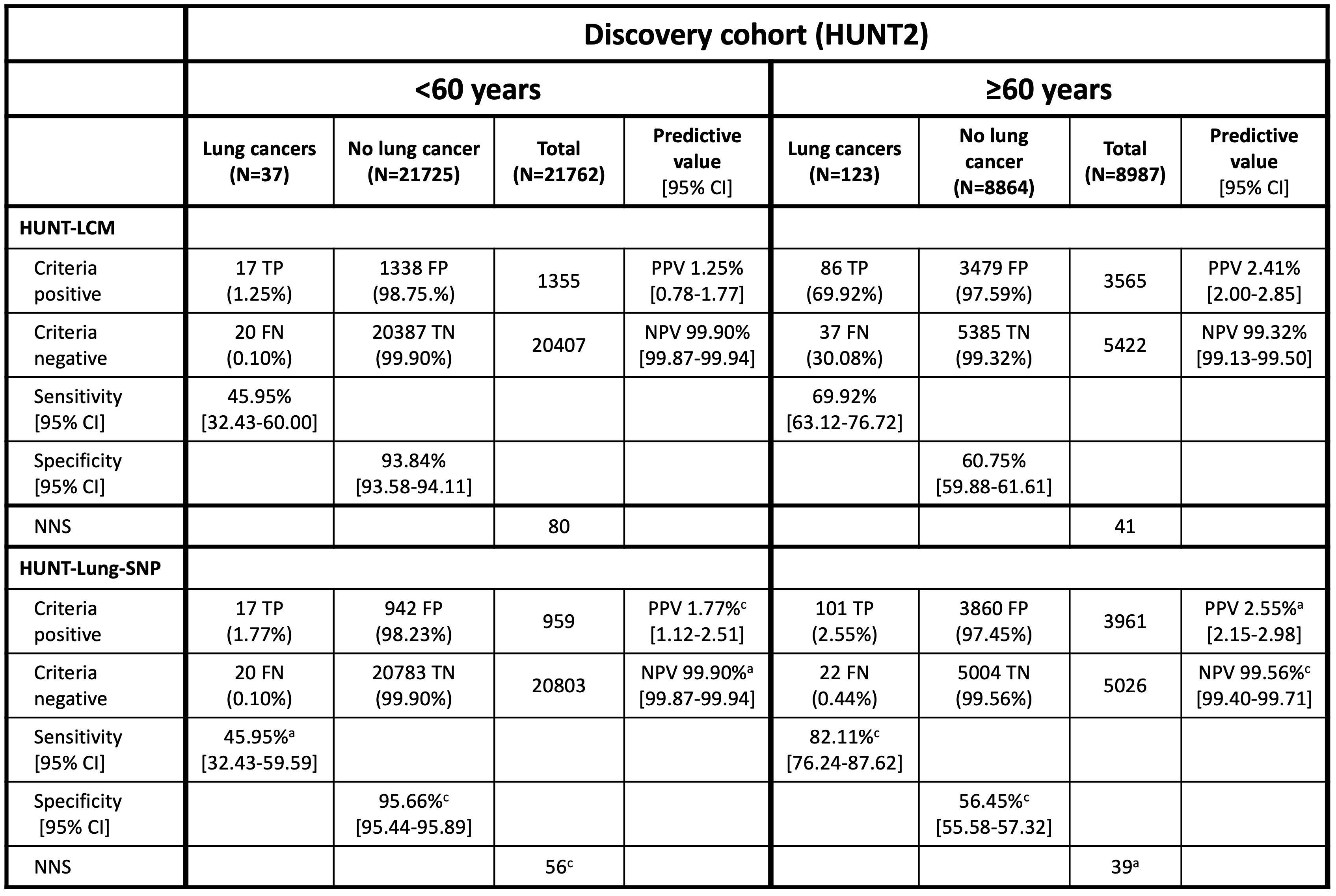
**

**Supplementary Table 6**. Comparison of the HUNT Lung-SNP and HUNT Lung Cancer Model (HUNT LCM) against the NLST criteria. The comparison is performed by considering the number of individuals selected by the NLST, i.e., n = 1233 on the HUNT2 discovery cohort (n=30749) and n = 101 on the Tromsø (n=2663) validation cohort.

FN, false negative; FP, false positive; NPV, negative predictive value; PPV, positive predictive value; TN, true negative; TP, true positive.

NLST criteria: at least 30 pack-years, at most 15 years quit time, age between 55 and 74 years old.

^a^p>0.05, versus NLST
^b^p<0.05, versus NLST
^c^p<0.01, versus NLST


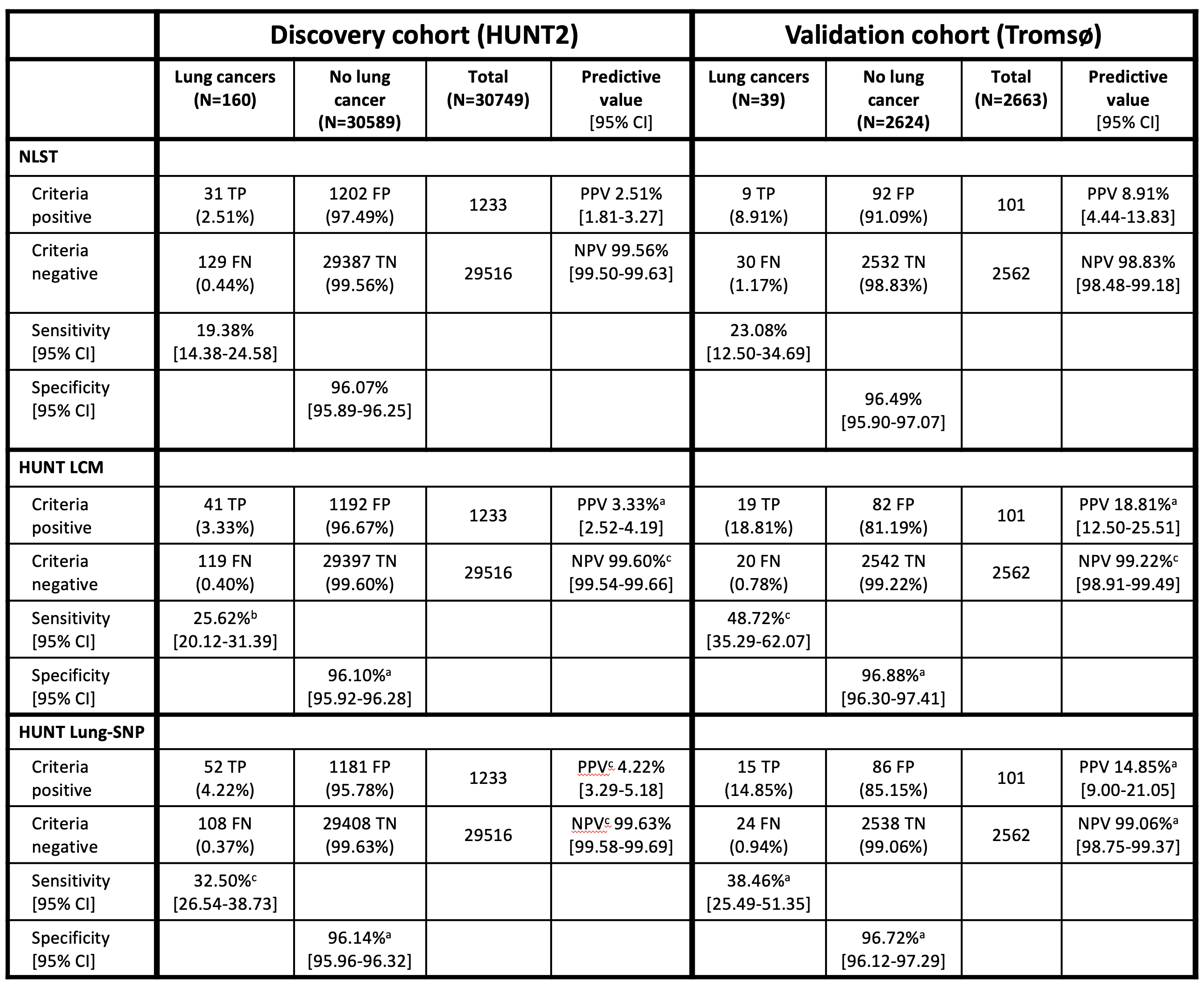


**Supplementary Table 7**. Comparison of the HUNT Lung-SNP and HUNT Lung Cancer Model (HUNT LCM) against the NELSON criteria. The comparison is performed by considering the number of individuals selected by the NELSON, i.e., n = 2324 on the HUNT2 discovery cohort (n=30749) and n = 206 on the Tromsø (n=2663) validation cohort.

NELSON criteria: Age between 50 to 74 years old, >15 cigarettes per day for >25 years or >10 cigarettes per day for >30 years, quit smoking ≤10 years. FN, false negative; FP, false positive; NPV, negative predictive value; PPV, positive predictive value; TN, true negative; TP, true positive.

^a^p>0.05, versus NELSON
^b^p<0.05, versus NELSON
^c^p<0.01, versus NELSON


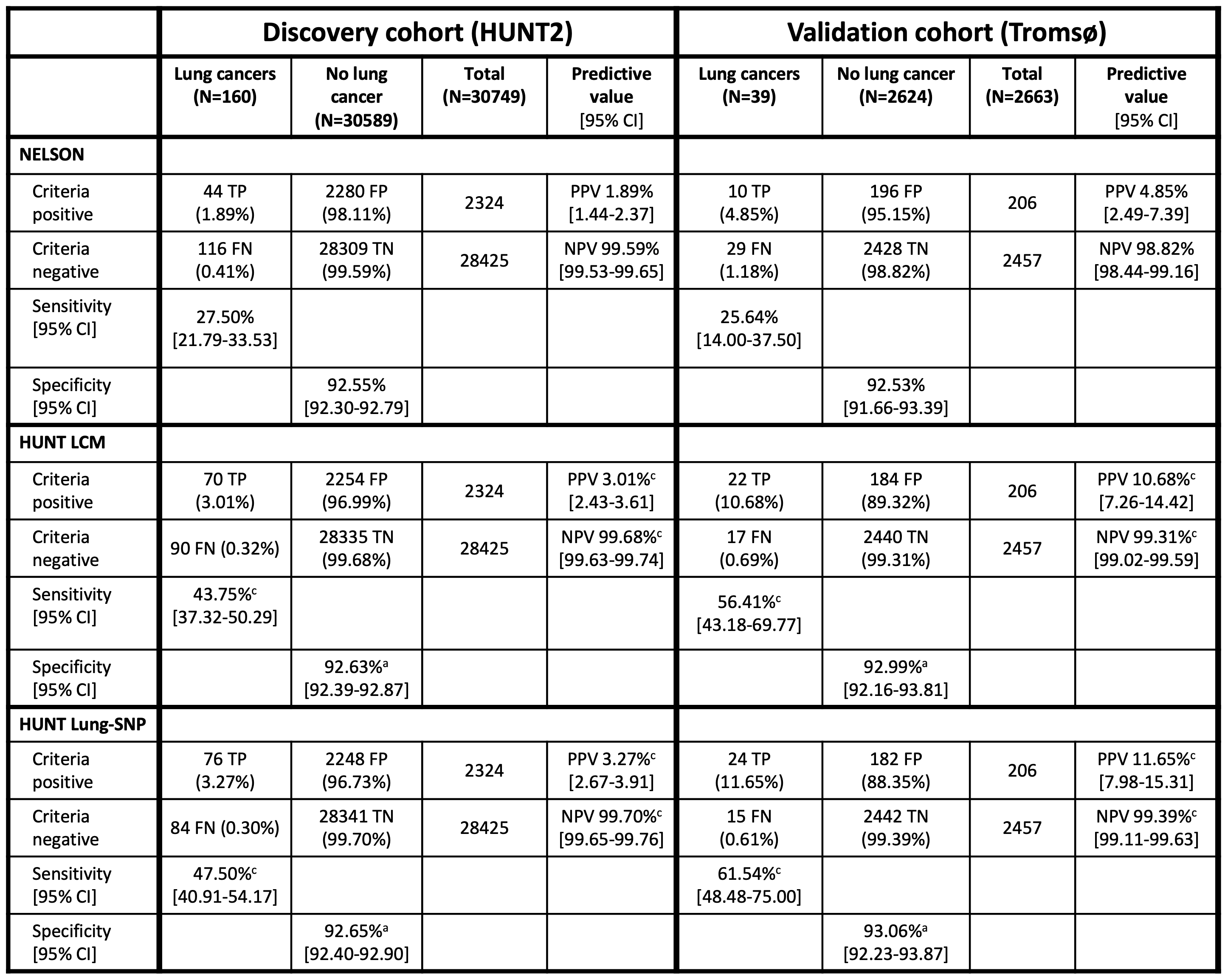


**Supplementary Table 8**. Comparison of the HUNT Lung-SNP and HUNT Lung Cancer Model (HUNT LCM) against the 2021 USPSTF criteria. The comparison is performed by considering the number of individuals selected by the 2021 USPSTF criteria, i.e., n = 4010 on the HUNT2 discovery cohort (n=30749) and n = 313 on the Tromsø (n=2663) validation cohort.

USPSTF criteria: age between 50 to 80 years old, at least 20 pack-years, and currently smoking or quit smoking < 15 years.

FN, false negative; FP, false positive; NPV, negative predictive value; PPV, positive predictive value; TN, true negative; TP, true positive.

^a^p>0.05, versus 2021 USPSTF
^b^p<0.05, versus 2021 USPSTF
^c^p<0.01, versus 2021 USPSTF


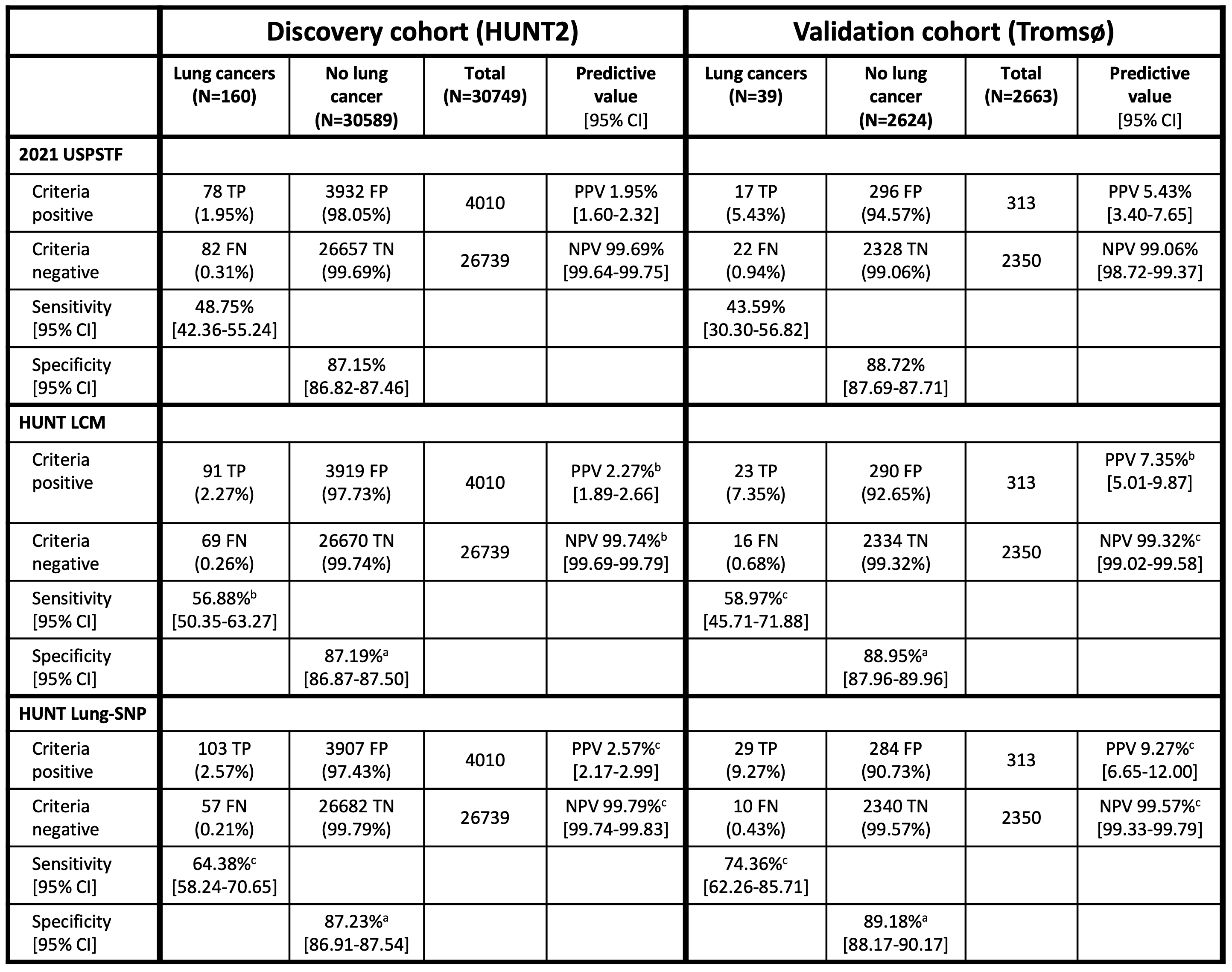


**Supplementary Table 9.** Characteristics of the 21 true high-risk ever-smokers predicted as high-risk for lung cancer within six years by the polygenetic HUNT Lung-SNP, but not by the clinical HUNT Lung Cancer Model (HUNT LCM) by analyzing the top 16^th^ percentile risk in six years. The age and the clinical variables are at inclusion in the HUNT study, not at lung cancer diagnosis.

| **Patient** | **Clinical variables** | | | | | | | | **22 SNPs** | **Risk score (%)** | |
| --- | --- | --- | --- | --- | --- | --- | --- | --- | --- | --- | --- |
|  | Sex | Age | Pack-years | Cough Daily | Smo ExpH | SmoDy CesDu | SmoCig DyN | BMI |  | Hunt Lung-SNP | Hunt LCM |
| 1 | 0 | 60.3 | 2 | 1 | 0 | 0 | 5 | 24.7 |  | 2.0942 | 0.2206 |
| 2 | 1 | 68.2 | 2 | 0 | 6 | 0 | 5 | 26.9 |  | 2.2563 | 0.2753 |
| 3 | 0 | 69.9 | 2 | 0 | 0 | 16 | 5 | 24.1 |  | 2.1033 | 0.1118 |
| 4 | 0 | 72.6 | 2 | 0 | 0 | 0 | 5 | 25.8 |  | 2.0797 | 0.2193 |
| 5 | 1 | 73 | 2 | 0 | 0 | 0 | 5 | 23.1 |  | 2.6826 | 0.3653 |
| 6 | 1 | 75.1 | 2 | 0 | 0 | 0 | 5 | 24.1 |  | 4.8942 | 0.3678 |
| 7 | 1 | 75.5 | 2 | 1 | 0 | 0 | 5 | 26.3 |  | 4.0082 | 0.5279 |
| 8 | 0 | 76.4 | 2 | 1 | 0 | 0 | 7 | 23.5 |  | 13.312 | 0.4411 |
| 9 | 1 | 80 | 2 | 1 | 0 | 0 | 8 | 21.1 |  | 16.298 | 0.7759 |
| 10 | 1 | 80.2 | 2 | 0 | 0 | 0 | 5 | 21.8 |  | 5.1232 | 0.5117 |
| 11 | 1 | 81.9 | 2 | 0 | 6 | 0 | 5 | 20.9 |  | 3.1363 | 0.6739 |
| 12 | 1 | 77.9 | 2,4 | 0 | 8 | 0 | 4 | 22.8 |  | 3.9878 | 0.6314 |
| 13 | 0 | 69.6 | 5,1 | 0 | 0 | 3 | 5 | 31.2 |  | 2.1147 | 0.2263 |
| 14 | 0 | 72 | 9,2 | 0 | 0 | 4 | 4 | 24.6 |  | 5.9686 | 0.6427 |
| 15 | 0 | 67.4 | 10 | 0 | 0 | 0 | 8 | 25.4 |  | 2.2120 | 0.6893 |
| 16 | 1 | 47.5 | 10,5 | 0 | 0 | 0 | 5 | 24 |  | 2.0430 | 0.3591 |
| 17 | 0 | 65.5 | 13 | 0 | 3 | 0 | 5 | 32.4 |  | 3.0770 | 0.7005 |
| 18 | 1 | 73.7 | 17,1 | 0 | 0 | 18 | 9 | 29.8 |  | 2.0538 | 0.7731 |
| 19 | 0 | 67.8 | 20 | 0 | 0 | 2 | 15 | 28 |  | 2.0073 | 0.7155 |
| 20 | 1 | 43.3 | 27 | 1 | 0 | 0 | 20 | 18.1 |  | 2.6480 | 0.9152 |
| 21 | 1 | 62.3 | 36,3 | 0 | 0 | 16 | 30 | 26.5 |  | 2.0000 | 0.5716 |

**Supplementary Table 10.** Key differences of the HUNT Lung-SNP model over other polygenic-clinical lung cancer risk prediction models. Most models lack external validation so far.

**
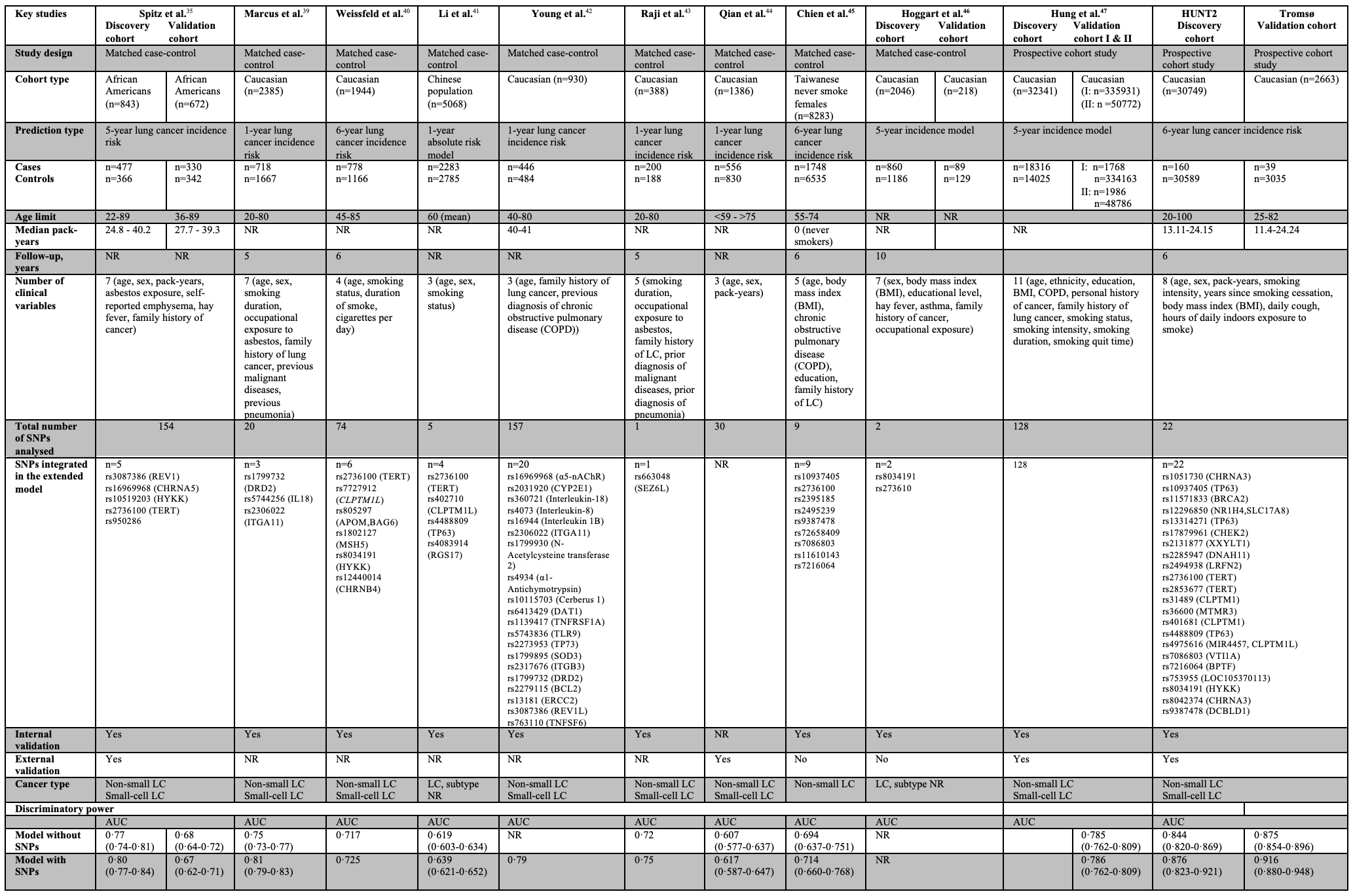
**

**Supplementary Table 11.** The demographic data of the genotyped individuals in the discovery (HUNT2) and validation cohort (Tromsø).

| **Clinical variables** | **Discovery cohort (HUNT2)**  **(N=30749)** | **Validation cohort (Tromsø)**  **(N=2663)** |
| --- | --- | --- |
| **Sex**  - Female  - Male | 14743 (47.9%)  16006 (52.1%) | 1355 (50.9%)  1308 (49.1%) |
| **Age**  - Mean (SD)  - Range | 51.298 (15.164)  20.2 - 100.3 | 50.174 (12.461)  25.0 - 82.0 |
| **Pack-years**  - Mean (SD)  - Range | 12.366 (11.381)  0.0 - 165.0 | 13.323 (12.426)  0.0 - 120.0 |
| **Daily cough parts of the year**  - No  - Yes | 24945 (81.1%)  5804 (18.9%) | 2164 (81.3%)  499 (18.7%) |
| **Indoor smoke exposure in hours**  - Mean (SD)  - Range | 2.070 (3.919)  0 - 24 | 2.766 (4.026)  0 - 24 |
| **Quit time in years**  - Mean (SD)  - Range | 6.716 (10.435)  0.0 – 75.0 | 6.113 (9.899)  0.0 – 76.0 |
| **Cigarettes daily**  - Mean (SD)  - Range | 11.110 (6.717)  1.0 - 70.0 | 11.668 (7.187)  0.0 -70.0 |
| **Body Mass Index (BMI)**  - Mean (SD)  - Range | 26.249 (4.018)  14.9-52.8 | 25.671 (3.755)  15.8 – 44.4 |

**Supplementary Fig. 1** Distribution of the 22 lung cancer associated SNPs according to geographical area and ethnicity (all references in Supplementary Table 1). The same color represents the same SNP. Among the 22 SNPs applied in our HUNT Lung-SNP model some have reported association with lung cancer only in Asian populations. These SNPs were informative in our combined HUNT Lung-SNP model in a predominantly Caucasian population.


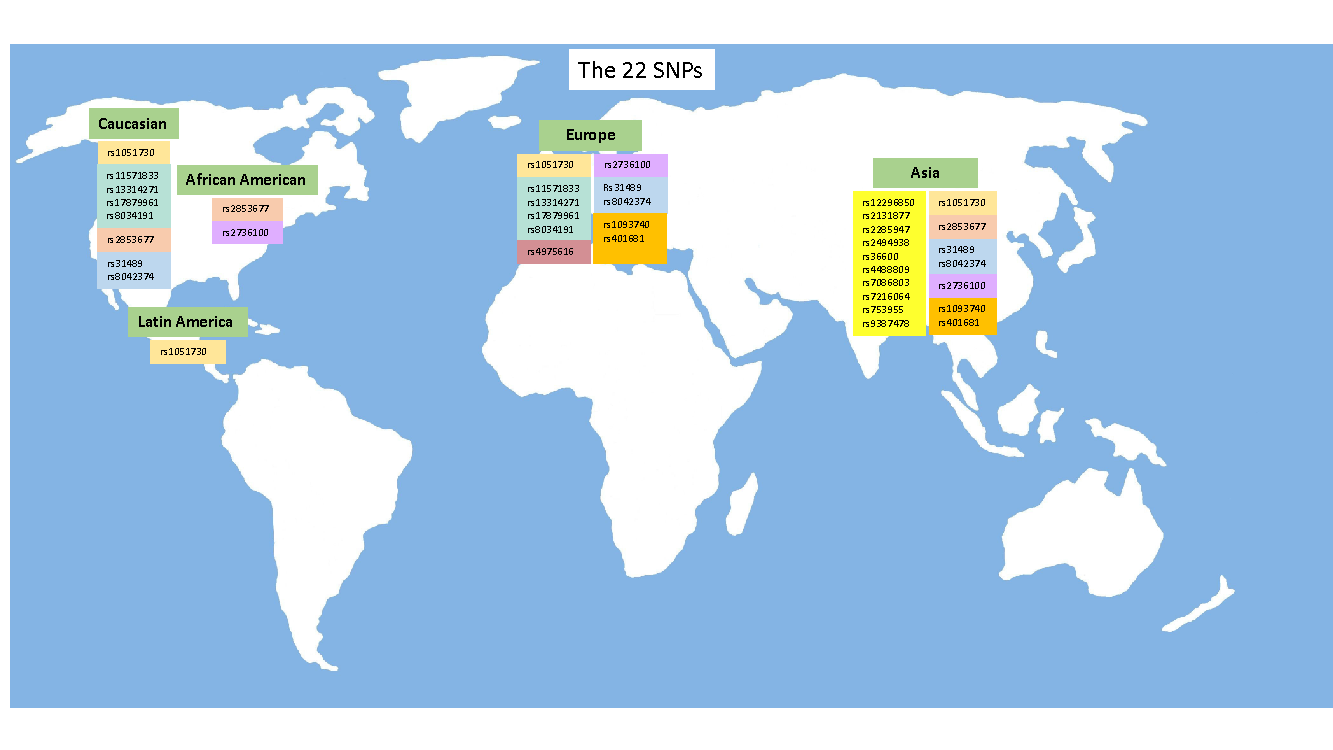


**Supplementary Fig. 2** Calibration plots of the HUNT Lung Cancer Model (HUNT LCM) and HUNT Lung-SNP model show the agreement between the models’ predicted risk and observed risk in the A) discovery cohort HUNT2 and B) validation cohort Tromsø. Log proportional linear models were used to estimate the expected risk within each of the five deciles and the average odds ratio of the observed and expected (predicted) risk over all the deciles were calculated. O/E, observed/expected ratio.


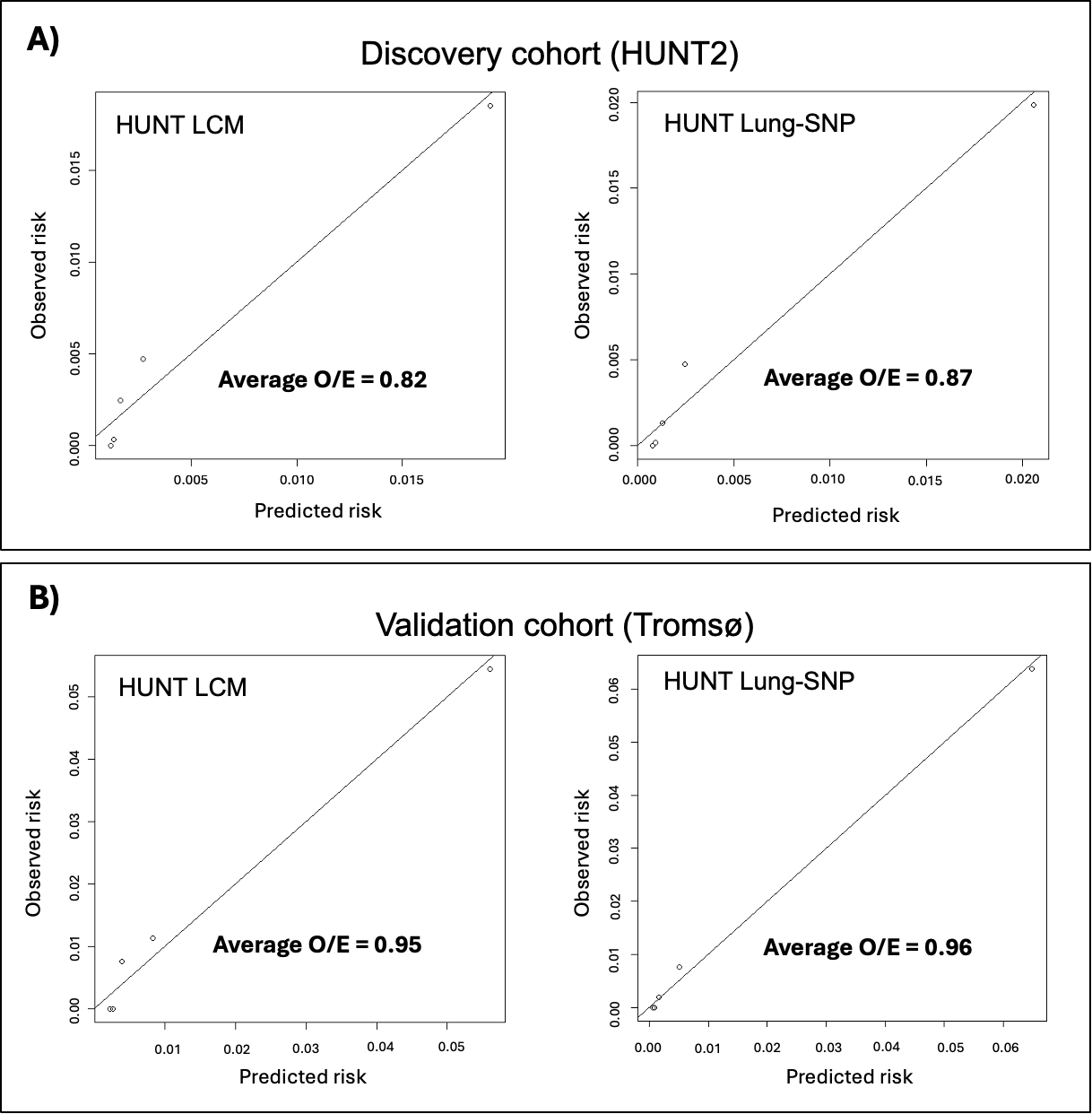


**Supplementary Fig. 3** Number of individuals needed to screen (NNS) to identify one case of lung cancer in the HUNT2 and Tromsø population. NNS computed when screening the same number of people selected by A) NLST criteria, B) NELSON criteria, C) 2021 USPSTF criteria.

NLST criteria: at least 30 pack-years, at most 15 years quit time, age between 55 and 74 years old.

NELSON criteria: >15 cigarettes per day for >25 years or >10 cigarettes per day for >30 years, age between 50 to 74 years old, quit smoking ≤10 years.

2021 USPSTF criteria: age between 50 to 80 years old, at least 20 pack-years, and currently smoking or quit smoking <15 years.

*p>0.05

**p<0.05

***p<0.01


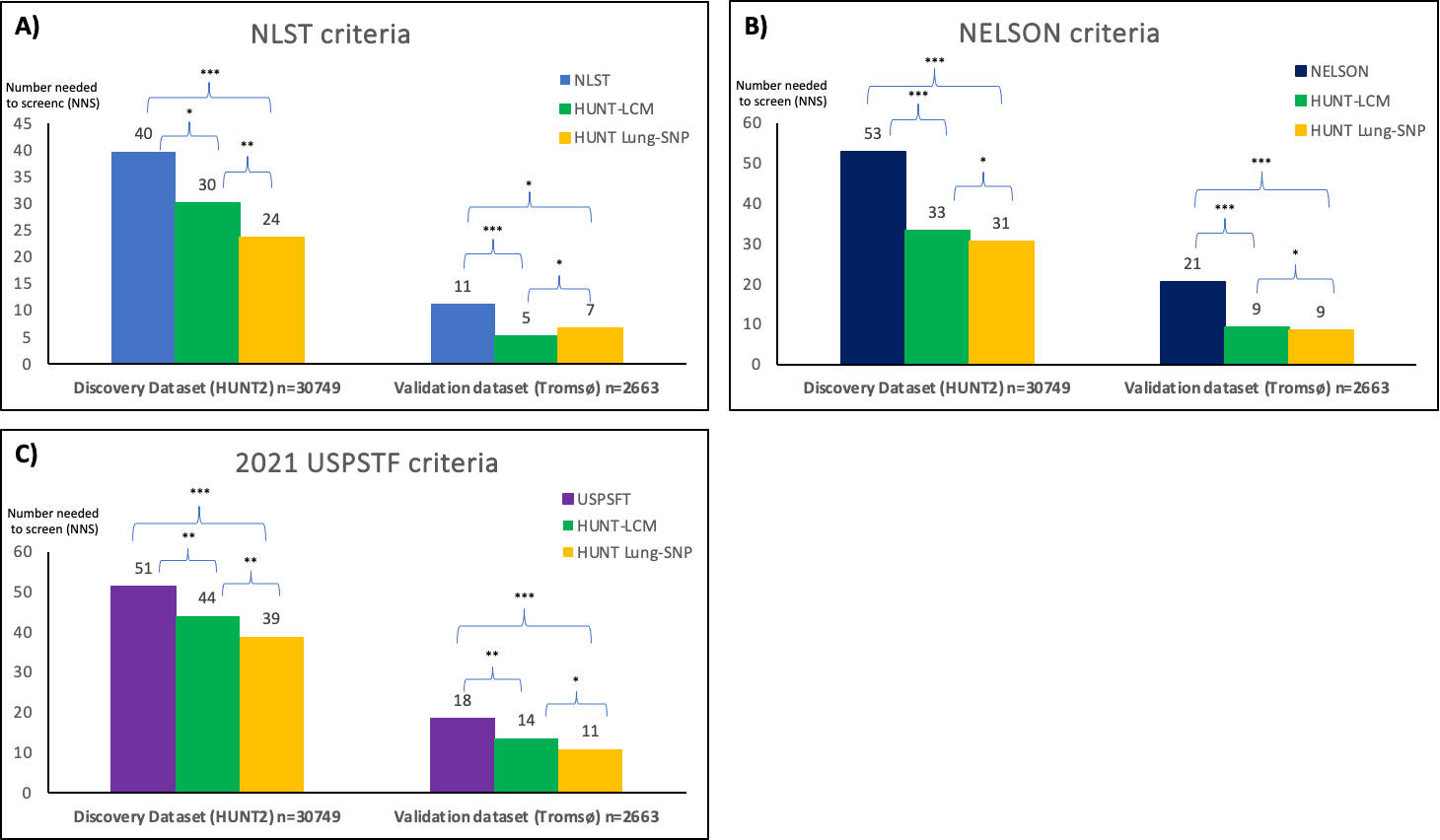


**Supplementary Fig. 4** Improving selection of individuals before CT screening. Cartoon comparing NLST, NELSON, USPSTF, the clinical HUNT LCM and the polygenic-clinical HUNT Lung-SNP model in the HUNT2 population of 30749 genotyped individuals, all ever-smokers, where 160 developed lung cancer in six years. The dark gray boxes depict diagnosed lung cancers within the six years. The timeline on the top depicts hypothetical screenings. CT, computer tomography; HUNT LCM, HUNT Lung Cancer model; HUNT Lung-SNP, HUNT covariates plus 22 SNP model

NLST criteria: at least 30 pack-years, at most 15 years quit time, age between 55 and 74 years old.

NELSON criteria: Age between 50 to 74 years old, >15 cigarettes per day for >25 years or >10 cigarettes per day for >30 years, quit smoking ≤10 years.

2021 USPSTF criteria: age between 50 to 80 years old, at least 20 pack-years, and currently smoking or quit smoking <15 years.


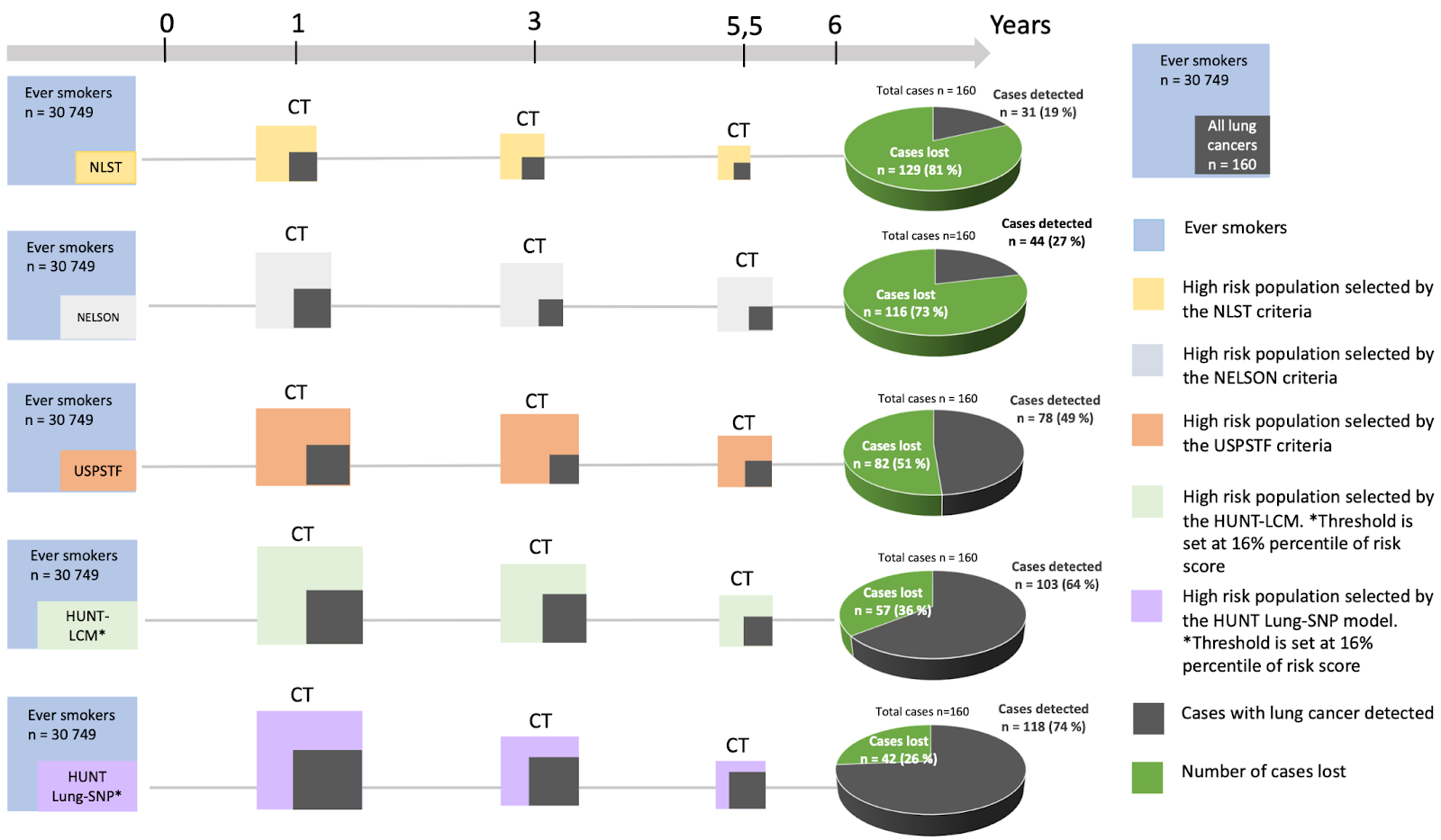


**Supplementary Fig. 5** Overall survival from the time of lung cancer diagnosis to death in HUNT2. Median survival is <12 months in all the groups. Insignificant differences in mortality amongst the HUNT Lung-SNP detected subjects in HUNT2 that developed lung cancer within six years compared to the true positive subjects within six years detected by NLST, NELSON and 2021 USPSTF criteria. Kaplan-Meier curves: A) HUNT Lung-SNP vs. NLST criteria. B) HUNT Lung-SNP vs. NELSON criteria. C) HUNT Lung-SNP vs. 2021 USPSTF criteria.


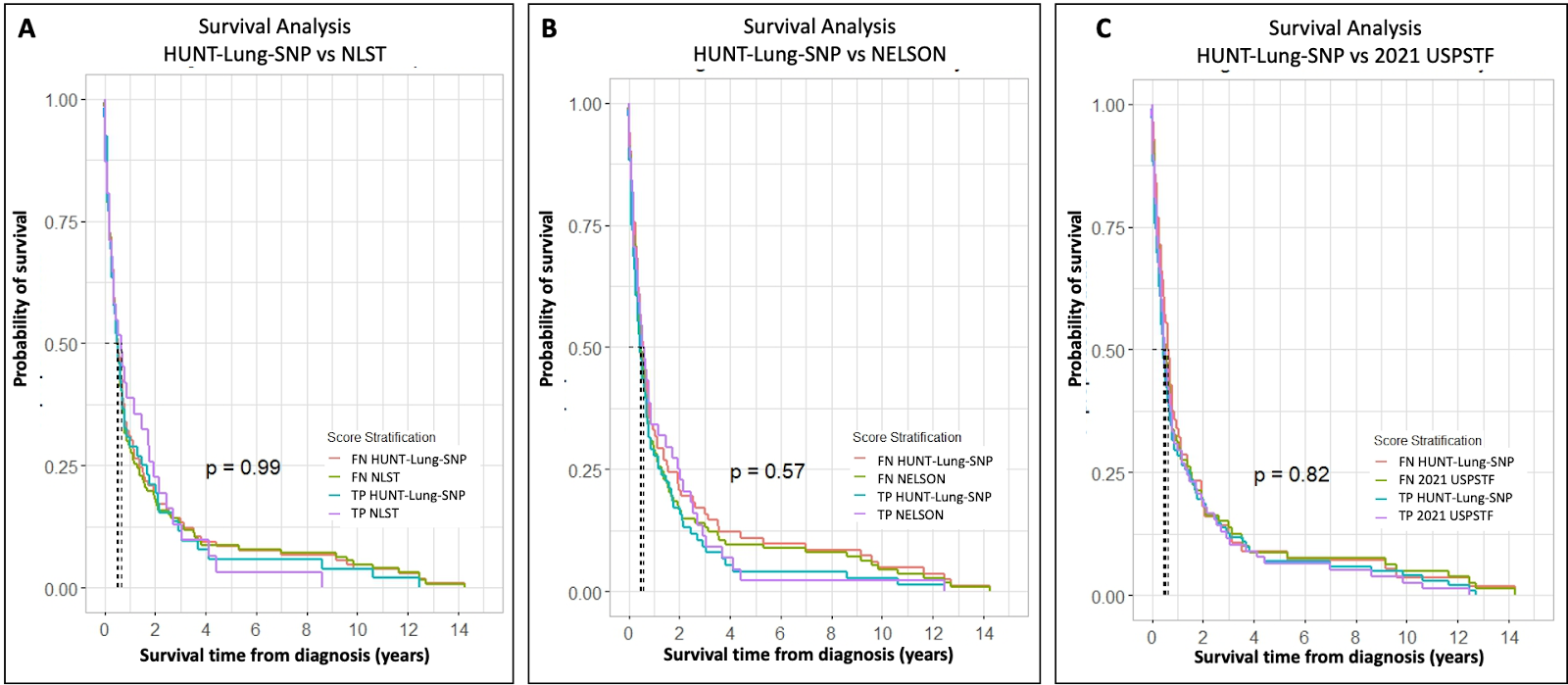


**Supplementary Fig. 6** Venn diagram describing the distribution of lung cancer cases within six years by the HUNT Lung-SNP model and the HUNT Lung Cancer Model (HUNT LCM) (top 16th percentile).


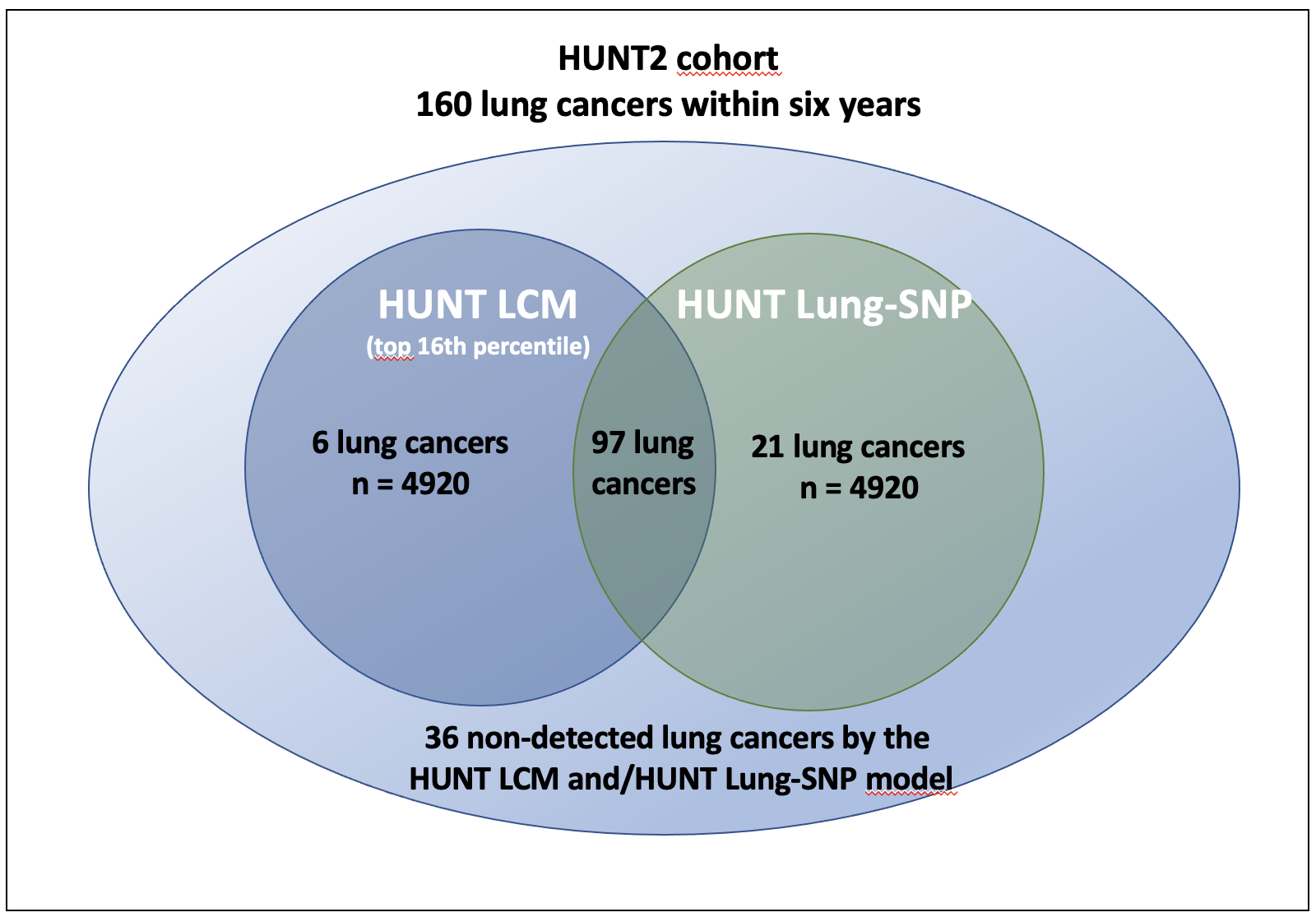


**References**

1. Das S, Forer L, Schönherr S, Sidore C, Locke AE, Kwong A, et al. Next-generation genotype imputation service and methods. Nature genetics. 2016;48(10):1284-7.

2. McCarthy S, Das S, Kretzschmar W, Delaneau O, Wood AR, Teumer A, et al. A reference panel of 64,976 haplotypes for genotype imputation. Nature genetics. 2016;48(10):1279-83.

3. Epidemiology NKGJCfG. ALL‐IN Fast‐track SNPs. Metadata-information sheet for SNP. Document version 2018‐06‐25. 2018.

4. HUNT Fast Track GWAS catalog [Internet]. NTNU/HUNT: https://www.ntnu.no/huntgenes/fasttrack. Available from: https://www.ntnu.no/huntgenes/fasttrack.

5. He D, Wang Z, Parida L. Data-driven encoding for quantitative genetic trait prediction. BMC Bioinformatics. 2015;16(1):S10.

6. Harrell FE, Jr., Lee KL, Califf RM, Pryor DB, Rosati RA. Regression modelling strategies for improved prognostic prediction. Stat Med. 1984;3(2):143-52.

7. Harrell FE, Jr., Lee KL, Mark DB. Multivariable prognostic models: issues in developing models, evaluating assumptions and adequacy, and measuring and reducing errors. Stat Med. 1996;15(4):361-87.

8. Steyerberg EW, Eijkemans MJC, Habbema JDF. Application of Shrinkage Techniques in Logistic Regression Analysis: A Case Study. Statistica Neerlandica. 2001;55(1):76-88.

9. Benjamini Y, Heller R. Screening for partial conjunction hypotheses. Biometrics. 2008;64(4):1215-22.

10. Illumina. Cost of Next-Generation Sequencing: Illumina; 2023 [cited 2023 15.09.23]. Available from: https://emea.illumina.com/science/technology/next-generation-sequencing/beginners/ngs-cost.html.

11. Brustugun OT, Møller B, Helland A. Years of life lost as a measure of cancer burden on a national level. British journal of cancer. 2014;111(5):1014-20.

12. Burnet NG, Jefferies SJ, Benson RJ, Hunt DP, Treasure FP. Years of life lost (YLL) from cancer is an important measure of population burden--and should be considered when allocating research funds. British journal of cancer. 2005;92(2):241-5.

13. lungekreft Nkf. Årsrapport 2022. 2023.

14. Behar Harpaz S, Weber MF, Wade S, Ngo PJ, Vaneckova P, Sarich PEA, et al. Updated cost-effectiveness analysis of lung cancer screening for Australia, capturing differences in the health economic impact of NELSON and NLST outcomes. British journal of cancer. 2023;128(1):91-101.

15. Zhan P, Song Y. CHRNA3 rs1051730 polymorphism and lung cancer susceptibility in Asian population: a meta-analysis. Translational lung cancer research. 2015;4(1):104-8.

16. Landi MT, Chatterjee N, Yu K, Goldin LR, Goldstein AM, Rotunno M, et al. A genome-wide association study of lung cancer identifies a region of chromosome 5p15 associated with risk for adenocarcinoma. American journal of human genetics. 2009;85(5):679-91.

17. Liu C, Cui H, Gu D, Zhang M, Fang Y, Chen S, et al. Genetic polymorphisms and lung cancer risk: Evidence from meta-analyses and genome-wide association studies. Lung cancer (Amsterdam, Netherlands). 2017;113:18-29.

18. Pérez-Morales R, González-Zamora A, González-Delgado MF, Calleros Rincón EY, Olivas Calderón EH, Martínez-Ramírez OC, et al. CHRNA3 rs1051730 and CHRNA5 rs16969968 polymorphisms are associated with heavy smoking, lung cancer, and chronic obstructive pulmonary disease in a mexican population. Ann Hum Genet. 2018;82(6):415-24.

19. Schwartz AG, Cote ML, Wenzlaff AS, Land S, Amos CI. Racial differences in the association between SNPs on 15q25.1, smoking behavior, and risk of non-small cell lung cancer. J Thorac Oncol. 2009;4(10):1195-201.

20. Wang Y, Broderick P, Webb E, Wu X, Vijayakrishnan J, Matakidou A, et al. Common 5p15.33 and 6p21.33 variants influence lung cancer risk. Nature genetics. 2008;40(12):1407-9.

21. He P, Yang XX, He XQ, Chen J, Li FX, Gu X, et al. CHRNA3 polymorphism modifies lung adenocarcinoma risk in the Chinese Han population. International journal of molecular sciences. 2014;15(4):5446-57.

22. Amos CI, Wu X, Broderick P, Gorlov IP, Gu J, Eisen T, et al. Genome-wide association scan of tag SNPs identifies a susceptibility locus for lung cancer at 15q25.1. Nature genetics. 2008;40(5):616-22.

23. Wang Y, McKay JD, Rafnar T, Wang Z, Timofeeva MN, Broderick P, et al. Rare variants of large effect in BRCA2 and CHEK2 affect risk of lung cancer. Nature genetics. 2014;46(7):736-41.

24. Hu Z, Wu C, Shi Y, Guo H, Zhao X, Yin Z, et al. A genome-wide association study identifies two new lung cancer susceptibility loci at 13q12.12 and 22q12.2 in Han Chinese. Nature genetics. 2011;43(8):792-6.

25. Miki D, Kubo M, Takahashi A, Yoon KA, Kim J, Lee GK, et al. Variation in TP63 is associated with lung adenocarcinoma susceptibility in Japanese and Korean populations. Nature genetics. 2010;42(10):893-6.

26. Wang Y, Broderick P, Matakidou A, Vijayakrishnan J, Eisen T, Houlston RS. Variation in TP63 is associated with lung adenocarcinoma in the UK population. Cancer epidemiology, biomarkers & prevention : a publication of the American Association for Cancer Research, cosponsored by the American Society of Preventive Oncology. 2011;20(7):1453-62.

27. Jin G, Ma H, Wu C, Dai J, Zhang R, Shi Y, et al. Genetic variants at 6p21.1 and 7p15.3 are associated with risk of multiple cancers in Han Chinese. American journal of human genetics. 2012;91(5):928-34.

28. Bae EY, Lee SY, Kang BK, Lee EJ, Choi YY, Kang HG, et al. Replication of results of genome-wide association studies on lung cancer susceptibility loci in a Korean population. Respirology. 2012;17(4):699-706.

29. Rafnar T, Sulem P, Stacey SN, Geller F, Gudmundsson J, Sigurdsson A, et al. Sequence variants at the TERT-CLPTM1L locus associate with many cancer types. Nature genetics. 2009;41(2):221-7.

30. Yoon KA, Park JH, Han J, Park S, Lee GK, Han JY, et al. A genome-wide association study reveals susceptibility variants for non-small cell lung cancer in the Korean population. Human molecular genetics. 2010;19(24):4948-54.

31. Broderick P, Wang Y, Vijayakrishnan J, Matakidou A, Spitz MR, Eisen T, et al. Deciphering the impact of common genetic variation on lung cancer risk: a genome-wide association study. Cancer research. 2009;69(16):6633-41.

32. Dong J, Jin G, Wu C, Guo H, Zhou B, Lv J, et al. Genome-wide association study identifies a novel susceptibility locus at 12q23.1 for lung squamous cell carcinoma in han chinese. PLoS Genet. 2013;9(1):e1003190.

33. Shiraishi K, Kunitoh H, Daigo Y, Takahashi A, Goto K, Sakamoto H, et al. A genome-wide association study identifies two new susceptibility loci for lung adenocarcinoma in the Japanese population. Nature genetics. 2012;44(8):900-3.

34. Wang Y, Broderick P, Matakidou A, Eisen T, Houlston RS. Role of 5p15.33 (TERT-CLPTM1L), 6p21.33 and 15q25.1 (CHRNA5-CHRNA3) variation and lung cancer risk in never-smokers. Carcinogenesis. 2010;31(2):234-8.

35. Spitz MR, Amos CI, Land S, Wu X, Dong Q, Wenzlaff AS, et al. Role of selected genetic variants in lung cancer risk in African Americans. J Thorac Oncol. 2013;8(4):391-7.

36. Lan Q, Hsiung CA, Matsuo K, Hong YC, Seow A, Wang Z, et al. Genome-wide association analysis identifies new lung cancer susceptibility loci in never-smoking women in Asia. Nature genetics. 2012;44(12):1330-5.

37. Van Dyke AL, Cote ML, Wenzlaff AS, Abrams J, Land S, Iyer P, et al. Chromosome 5p Region SNPs Are Associated with Risk of NSCLC among Women. J Cancer Epidemiol. 2009;2009:242151.

38. Zanetti KA, Wang Z, Aldrich M, Amos CI, Blot WJ, Bowman ED, et al. Genome-wide association study confirms lung cancer susceptibility loci on chromosomes 5p15 and 15q25 in an African-American population. Lung cancer (Amsterdam, Netherlands). 2016;98:33-42.

39. Marcus MW, Raji OY, Duffy SW, Young RP, Hopkins RJ, Field JK. Incorporating epistasis interaction of genetic susceptibility single nucleotide polymorphisms in a lung cancer risk prediction model. International journal of oncology. 2016;49(1):361-70.

40. Weissfeld JL, Lin Y, Lin HM, Kurland BF, Wilson DO, Fuhrman CR, et al. Lung Cancer Risk Prediction Using Common SNPs Located in GWAS-Identified Susceptibility Regions. J Thorac Oncol. 2015;10(11):1538-45.

41. Li H, Yang L, Zhao X, Wang J, Qian J, Chen H, et al. Prediction of lung cancer risk in a Chinese population using a multifactorial genetic model. BMC medical genetics. 2012;13:118.

42. Young RP, Hopkins RJ, Hay BA, Epton MJ, Mills GD, Black PN, et al. A gene-based risk score for lung cancer susceptibility in smokers and ex-smokers. Postgrad Med J. 2009;85(1008):515-24.

43. Raji OY, Agbaje OF, Duffy SW, Cassidy A, Field JK. Incorporation of a genetic factor into an epidemiologic model for prediction of individual risk of lung cancer: the Liverpool Lung Project. Cancer prevention research (Philadelphia, Pa). 2010;3(5):664-9.

44. Qian DC, Han Y, Byun J, Shin HR, Hung RJ, McLaughlin JR, et al. A Novel Pathway-Based Approach Improves Lung Cancer Risk Prediction Using Germline Genetic Variations. Cancer epidemiology, biomarkers & prevention : a publication of the American Association for Cancer Research, cosponsored by the American Society of Preventive Oncology. 2016;25(8):1208-15.

45. Chien LH, Chen CH, Chen TY, Chang GC, Tsai YH, Hsiao CF, et al. Predicting Lung Cancer Occurrence in Never-Smoking Females in Asia: TNSF-SQ, a Prediction Model. Cancer epidemiology, biomarkers & prevention : a publication of the American Association for Cancer Research, cosponsored by the American Society of Preventive Oncology. 2020;29(2):452-9.

46. Hoggart C, Brennan P, Tjonneland A, Vogel U, Overvad K, Østergaard JN, et al. A risk model for lung cancer incidence. Cancer prevention research (Philadelphia, Pa). 2012;5(6):834-46.

47. Hung RJ, Warkentin MT, Brhane Y, Chatterjee N, Christiani DC, Landi MT, et al. Assessing Lung Cancer Absolute Risk Trajectory Based on a Polygenic Risk Model. Cancer research. 2021;81(6):1607-15.
